# Abstracts of papers presented at the 1994 Pittsburgh Conference

**DOI:** 10.1155/S146392469400009X

**Published:** 1994


Journal of Automatic Chemistry, Vol. 16, No. 3 (May-June 1994), pp. 75-116

### Abstracts of papers presented at the 1994 Pittsburgh Conference

*The following are the abstracts of the papers read at March 1994s Pittcon which are important to readers of 'Journal of Automatic Chemistry'. 1995s Pittcon will be held in New Orleans from 5 to 10 March. Details from The Pittsburgh Conference, 300 Penn Center Boulevard, Suite 332, Pittsburgh, PA 15235-5503, USA.*

### Reflecting on the past; creating for the future

*Howard V. Malmstadt, University of the Nations, Kailua-Kona, HI 96740*

Powerful and elegant analytical methods and instruments are used daily in industry, hospitals, R & D laboratories and process control systems. Major improvements in sensitivity, detectability, accuracy, speed and reliability provide the necessary data for dramatic breakthroughs in science and technology. The speaker reflected on some of the developments on which he had helped pioneer.

The story behind the story often starts with a simple question or comment. Why wouldn't this work? There must be a better way! What would be the 'ideal' way? It takes too much time to do it that way! How can we make it available to others? Several examples were cited in which such questions and comments started the author on a search for answers and subsequently resulted in improved analytical methods and instrumentation.

There is a powerful incentive to learn and to find effective ways to do things when it is 'a matter of life and death'. Likewise, projects that are especially relevant and serve others tend to motivate. Research projects and course developments that have been influenced significantly by these considerations were described.

Modern microcomputer-based analytical instruments often provide push-button analyses. However, it is also important to master these new instruments, to understand the data flow and transformations that yield measurements and control our systems. It was shown how a small investment of time and effort can provide a sound, working knowledge of the new instruments, and how this experience can improve on-the-job efficiency and lead to creative solutions for projects.

### Highways and byways in mass spectrometry

*Klaus Biemann, Department of Chemistry, Massachusetts Institute of Technology, Cambridge, MA 02139-4307*

The author began his academic career in 1957 without any formal training in either analytical chemistry or mass spectrometry, but as a synthetic organic chemist with an interest in the determination of the structure of natural products. Therefore, no preconceived notions prevented him from applying mass spectrometry to the structure elucidation of new alkaloids, amino acids and peptides. A particularly productive field was the determination of the structure of many indole alkaloids. By classical chemical methods this was difficult and very time consuming, but with the use of mass spectrometry combined with a few chemical reactions, the task could be accomplished quickly and with minimal material. Thus, the early work brought mass spectrometry to the attention of the organic chemist.

In the 1960s, high resolution mass spectrometry was introduced to organic and biochemistry, and an interface between a gas chromatograph and a mass spectrometer (GC-MS) was developed. The large amount of data generated by these two techniques required the use of computers and the development of appropriate algorithms, as early as 1963. One of the latter, which permitted the identification of an unknown by automatic comparison of its mass spectrum with a library of spectra of known compounds, gained wide acceptance and, after improvements by others, is still in use.

The author's experience in GC-MS and the computer-aided interpretation of the resulting mass spectra led to his leading role in the search for organic compounds in the surface materials of the moon (Apollo 11, 12 and 14) and Mars (Viking, 1976). Nevertheless, his early interest in the development of mass spectrometric methods for the determination of the amino acid sequence of peptides, and finally proteins, continued throughout this time, which became a practical alternative to the classical Edman degradation in the late 1970s. At first, it involved a series of chemical reactions to convert the peptides to derivatives sufficiently volatile for GC-MS. However, when it became possible to directly ionize underivatized peptides, he developed protein sequencing methodology by high-energy collision-induced-dissociation tandem mass spectrometry.

Since its inception 35 years ago, the author's work was greatly aided by the about 135 graduate students, postdoctoral fellows and visiting scientists whom he trained in mass spectrometry. They in turn, together with their numerous students and associates, continue to contribute to the field.

### The determination of mercury at ultratrace concentration

*Steven Jankowski, GBC Scientific Equipment Inc, 3930 Ventura Drive, Suite 355, Arlington Heights, IL 60004, Richard J. Gill and Thomas Gerdei, GBC Scientific Equipment Pty Ltd, PO Box 1226, Dandenong, Victoria 3175, Australia*

Continuous flow cold vapour mercury generation apparatus coupled to an atomic absorption spectrophotometer is able to achieve detection limit and characteristic concentration values of 0·05 and 0·3 μg Hg/litre respectively. A system which is able to improve sensitivity by an order of magnitude or better is required to comply with environmental legislation for mercury pollution. The combination of atomic absorption with a suitable mercury-concentration accessory device attached to the vapour generation apparatus would be desirable because of enhanced sensitivity, and its ability to be utilized for automated routine measurement. Concentration of mercury vapour can be achieved by entrapment onto a gold ribbon to form a gold–mercury amalgam. After mercury collection it is stripped from the gold surface by a controlled heating process. The atomic mercury vapour is then transferred to a quartz cell for atomic absorption measurement. The process produces a transient mercury signal that is proportional to the collection time. The combination of hardware with suitable data collection software allows for the total automation of sample presentation, mercury collection and signal measurement.

The enhanced sensitivity of the technique means that the contamination effects of reagents has had to be eliminated. The combination of software and design features to prevent this was discussed, along with presentation of data relating to the enhanced sensitivity and detection limit achievable for environmental samples.

### The determination of mercury in environmental samples using the Perkin–Elmer flow injection mercury system

*S. McIntosh, J. Baasner and C. Hanna, Perkin–Elmer Corporation, 761 Main Avenue, Norwalk, CT 06859-0219*

The determination of mercury in environmental samples can be improved when the flow injection technique is used. A procedure for the determination of mercury in environmental samples using a dedicated Flow Injection Mercury System (FIMS) was discussed. The flow injection technique technique automates the generation of cold vapour mercury providing increased sample throughput with reduced sample and reagent consumption. The kinetic discrimination of the flow injection technique reduces interference effects commonly associated with the traditional batch method, while also eliminating memory effects associated with the continuous flow method.

The FIMS consists of a flow injection system integrated with a low pressure mercury source, an electrically heated quartz cell and a solar-blind detector with optimum sensitivity at 253·7 nm. This compact system is completely controlled using an industry standard personal computer and advanced AA WinLab system software running under Microsoft Windows. The analysis of environmental samples is performed using the EPA approved Flow Injection Method 245.1A, in which the generation of the mercury vapour is accomplished using the flow injection system. The mercury vapour is swept into the electrically heated quartz cell and the absorption signal is measured. The length of the absorption cell has been optimized to provide maximum sensitivity resulting in a detection limit of mercury in drinking waters of less than 10 ng/l Hg. This represents a 20-fold improvement in the mercury detection limit previously obtained with the FIAS system. Maximum sample throughput is obtained by coupling the FIMS with an autosampler, resulting in a sample analysis rate equal to 120 determinations per hour.

The mercury detection limit can be further improved by using an amalgam accessory. The amalgamation technique preconcentrates mercury onto a gold gauze, which is heated to liberate the collected mercury. This process decouples the generation of the mercury cold vapour from the detection of the mercury, resulting in fewer matrix interferences. Because the mercury signal is dependent on the volume of mercury collected, the detection limit is defined by the sample volume available.

### The development of a simple trap to remove acetate interference for mercury determination in TCLP leachates

*David J. Hassett and Jeffrey S. Thompson, Energy & Environmental Research Center, University of North Dakota, Grand Forks, ND 58202*

The determination of mercury in toxicity characteristic leaching procedure (TCLP) leachates presents special problems for many commercially available mercury analysis systems. The presence of organic components in the determination of mercury in water samples causes a positive interference at the 253-nm absorption line used for cold vapour mercury analysis. Even with the commonly used acidic-permanganate-persulphate digestion for the determination of total mercury, much of the acetate used in TCLP leachates remains and can cause severe problems. The use of a simple soda lime trap to remove acetate interference in the analysis of mercury has been successfully demonstrated. The soda lime trap employed was constructed out of materials supplied by the manufacturer of the commercial analyser for use as a water trap and was simple to maintain. It was found that the useful lifetime of this trap paralleled that of the water trap, thus routine maintenance consisted of regular replacement of both traps on the same schedule. Additionally, the use of dual detection systems, such as cold vapour absorption and atomic fluorescence, has been employed to successfully identify the presence of this interference in routine determinations and also to provide a means of monitoring the efficiency of this system to avoid problems with saturated or inefficient traps.

### Online analysis of total mercury levels in plant applications by atomic fluorescence

*Peter B. Stockwell, Warren T. Corns, P S Analytical Ltd, Arthur House, B4 Chaucer Business Park, Watery Lane, Kemsing, Sevenoaks, Kent TN15 6QY, UK and J. Cloud, Spectra France, 5 Chemin des Carrieres, BP 33, 60340 Saint Leu d'Esserent, France*

The application of atomic fluorescence coupled to vapour generation techniques for the determination of mercury at ppt levels has been well established in laboratory applications. The detector configuration is ideally suited to process applications, being of simple design and rugged construction; in addition, the laboratory systems are configured using continuous flow principles. Operating the system on a continuous online basis, however, produces several different design constraints. First, the measurement is for total mercury so all the mercury species must be converted directly online prior to measurement. Second, the reagent addition must be matched to the rate of analyses and the need for these reagents to be stable for long periods.

Several chemical regimes have been examined and these can be matched to the sample input to optimize overall performance and reliability. For example a reverse flow injection system has been configured which uses the sample as the transport medium. Reagents are added to this to convert the organic mercury compounds into mercury and also stannous chloride to release the vapour to the detector. The analytical system is set up to provide analyses of the sample on a regular cycle. Check standards and blanks cann be exchanged for the sample input under computer control at selected times.

In addition, the standard bromate/bromide digestion approach used in laboratory applications has also been used in a standard discrete sample injection mode. The advantages and disadvantages of these chemical regimes were outlined.

The online system provides the sensitivity of the laboratory unit while minimizing servicing and reagent requirements. The data provided by each measurement can subsequently be sent to a process control computer, so that changes in the measurement levels can be corrected or adjusted as desired.

### Atomic fluorescence/gold amalgamation techniques for the determination of environmental mercury

*William R. Kammin, Randy Knox, James Ross and David Thomson, Washington State Department of Ecology, Manchester Environmental Laboratory, 7411 Beach Drive East, Port Orchard, WA 98366*

Water quality criteria have been promulgated by USEPA for the prevention of harm to natural environments. For total mercury, the chronic and acute criteria are in the low nanogram/litre range. The currently approved USEPA methods, which use the cold vapour atomic absorption method for detection, do not allow reliable quantitation in this range. In addition, the current digestion technique is slow, and requires large volumes of sulphuric and nitric acids. There are also significant problems with mercury contamination of the reagents used in the digestion procedure.

This presentation reported on the development of a new method, which combines atomic fluorescence, microwave sample preparation, and the use of a gold amalgamation/preconcentration technique.

The method produces full scale mercury response at 10 nanograms/litre. This response indicates suitability for ultra trace mercury determination. The method was evaluated using EPA quality control procedures called out in EPA method 245.1, 245.5, and the SW-846 series of mercury methods. These techniques include procedural blanks, spiked samples, duplicates, and the evaluation of instrument and method detection limits. Optimized instrument detection limits for the technique are in the parts per quadrillion range. Real world detection levels will be limited by mercury in the analytical blank.

### The use of atomic fluorescence detectors as specific detectors for mercury, arsenic and selenium speciation

*Peter B. Stockwell and Warren T. Corns, P S Analytical Ltd, Arthur House, B4 Chaucer Business Park, Watery Lane, Kemsing, Sevenoaks, Kent TN15 6QY, UK*

Speciation of mercury, arsenic and selenium is becoming increasingly important. Levels of these compounds, however, are exceedingly low and currently available techniques are often wanting in levels of performance. The atomic fluorescence detectors developed for coupling to vapour generation techniques have the sensitivity enhancement suitable for chromatographic systems. This paper will describe the application of the PSA Merlin and Excalibur Detectors to speciation studies.

The application of the Merlin and Excalibur atomic fluorescence detectors was described.

Several analytical schemes have been developed for mercury analysis. Initially the work used a purge and trap configuration linking the Merlin Detector to the column output using a simple pyrolysis interface. The output from the column is passed through a quartz furnace interface held at 800°C. The same interface has also been linked to the output of a capillary gas chromatographic column. A further study was reported where the output of an HPLC column is linked through a hydride generation interface to the Merlin Detector. Methods of separation and the chemical digestion and vapour generation were described.

In addition, speciation studies on arsenic and selenium were outlined using purge and trap techniques prior to vaporization of the hydrides formed for arsenic and selenium into a properly configured Excalibur Detector.

### Intelligent automation of the EPA QA/QC procedure for inorganic analyses

*Kenneth L. Seace, Diane Platt and Suzanne G. Hurt, Hitachi Instruments, Inc., 44 Old Ridgebury Rd, Danbury, CT 06801*

The EPA QA/QC procedure for analysing CLP inorganics is intricate, with different protocols mandated in response to different concentrations of analyte determined in each experiment. The protocol in this procedure is very specific in terms of the analytical sequence, with any deviation from the protocol invalidating the assay, and requiring it to be repeated. Hence, an experienced analyst must continually monitor the results from each assay and determine the correct course of subsequent action. This need for immediate scrutiny of each measurement adds to the stress and strain on the analyst in a busy laboratory. Useful automation of analyses should go beyond sample preparation and sample introduction into the instrument.

An automated system (Environmental Sampling Protocol) is now available that utilizes artificial intelligence to direct the atomic absorption spectrophotometer to exercise the necessary actions mandated, based on results from standards, samples, and related QC measurements, all without operator intervention. A full description of this system was presented.

### Using a windows-based database for review and management of HPLC data acquisition system results

*Steve Maykowski and Bob Giuffre, Hewlett–Packard Company, West 120 Century Road, Paramus, NJ 07653-0099*

Modern analytical instrumentation equipped with autosamplers generate vast amounts of information. Therefore, there is a need to organize this data for archival as well as review purposes. While the software that runs the analytical equipment is optimized for data acquisition and analysis, it usually falls short of the organization and archival needs of the user.

Information stored in a database can be presented to the user more quickly and in a way that allows the user to better understand the information that the system generates.

The relational database engine chosen for the application is Access from Microsoft. The instrumentation is a Hewlett–Packard 1090 HPLC system, running a Windows-based data acquisition software package.

This paper showed how information from many runs can be sorted and viewed. Among the capabilities is the ability to search the database for a specific peak name and have control charts automatically created. Since the user may want to view the chromatography, graphics from the data acquisition system are embedded in the database for viewing. This presentation can be set up with a timer which allows for automatic scrolling through all the files found in the search with no user intervention.

Additionally, column performance parameters are stored within the database. This information allows the user to track column performance information and to determine if system suitability protocols are within limits.

### Automatic, interactive system suitability in a PC-based HPLC system

*Terrance A. Rooney, Landy White and Charley Chell, Thermo Separation Products, 45757 Northport Loop West, Fremont, CA 94537*

System suitability is a widely used procedure in the pharmaceutical industry which determines whether an HPLC system is functioning properly and is suitable for collecting data which can be submitted to regulatory agencies. At the present, many system suitability calculations are done manually, introducing potential errors and poor reproducibility because of operator-to-operator variations in the measurements.

This paper described a new PC-based HPLC system which completely automates the system suitability procedures, removing operator variation and producing results which are completely documented and ready for submission to appropriate government agencies.

The software provides extensive high resolution, colour graphics of each peak of interest, showing in multiple colours the peak width at 50%, 10%, 5% and baseline, in addition to calculating the resolution, theoretical plates, USP (US Pharmacopoeia) Tailing Factor, EP (European Pharmacopoeia) Symmetry Factor, CFR (Code of Federal Regulations) Asymmetry Factor and various other measures used in system suitability.

The software fully automates the system testing process, allowing the user to choose which performance indicators on which to do the suitability measurements, as well as automatically taking the appropriate action in the case of non-compliance. Each parameter (such as retention time, tailing factor, resolution, theoretical plates) can have a minimum, a maximum and an RSD (relative standard deviation) setpoint. The user can also select number of re-tries in the event system is found not suitable, as well as the action to take if the system is still not suitable after the selected number of re-tries. Extensive GLP documentation is provided including time and data stamps of all files used, extensive notes on both the system configuration and sample being run, all column parameters, and the ROM levels and serial numbers of all instruments being used. The software's acquisition log automatically monitors the column temperature and pressure, and the baseline noise and drift, and saves them along with the raw data so the user has a complete record of events which occurred during the analysis.

### Software design considerations for a new approach to HPLC system communication

*Der-Min Fan and James A. Schibler, Dionex Corporation, 1228 Titan Way, Sunnyvale, CA 94088-3603*

The design of a new family of HPLC modules incorporates a unique approach to high speed bi-directional communications. Implementation of an Ethernet communications standard within the module allows the host PC to address up to eight systems, each with up to eight modules.

The new design supports high-level communication between the modules and the host PC. For example, the host can get information about each module, such as module type, firmware version, and operational status, simply by polling the network. If a module requires a firmware update, the new code can be downloaded directly across the instrument network, eliminating the need to open the module. Before the start of each run, the host can confirm that all required modules are present and ready, and all module information can be recorded with each raw data set to ensure traceability of results.

To implement the new design, several important issues had to be addressed. Each module needed a unique identifier embedded in its electronics so that modules of the same type could be discerned by the host PC. A convenient means of grouping the modules into systems had to be provided within the context of the graphical user interface. Decisions had to be made about how each module would keep track of time—absolute elapsed—and how the internal clocks in the modules would be synchronized.

Another set of issues revolved around the definitions of user access and instrument control. Three modes were implemented: Local (control at the module front panel), Remote (control from the host PC, with possibility to override at the front panel), and Remote Locked (control exclusively from a pre-written method on the host PC). The latter prevents pre-defined method parameters from being altered during a run, ensuring traceability of results as results as required by Good Laboratory Practices.

This paper also described software design considerations related to supporting this new family of HPLC modules.

### Automated HPLC spectral analysis reports

*Terrance A. Rooney and Christopher Crandell, Thermo Separation Products, 45757 Northport Loop West, Fremont, CA 94537*

The use of spectral information in HPLC analyses offers a number of significant advantages in quality assurance as well as to the methods development.

In the QA environment, routine use of detectors such as PDA (Photo Diode Array) and rapid scanning (such as the TSP FOCUS) provide spectral information which significantly increases reliability of peak purity results. In methods development, scanning detectors provide not only information about peak purity but also can provide positive peak identification through spectral matching with known compounds in a spectral library.

This paper described some recent enhancements to peak purity analyses, resulting in more reliable data even as low as the 0·1% concentration generally used in the pharmaceutical industry as the level of desired detection of impurities.

New software provides the user with the ability to specify both the peak threshold used for the purity analyses, as well as the peak coverage (up to 100%). User selection of peak coverage permits the chemist to optimize the peak purity algorithm depending on the signal to noise situation. If the signal to noise is relatively poor, a more limited peak coverage should be employed to avoid problems with doing correlation calculations at the peak extremities. If the signal to noise is high (as is the case in many routine pharmaceutical applications), the peak coverage can be increased to 100%, ensuring that impurities at the peak extremities will be detected.

A peak purity profile is printed which shows a cross section of the peak in the time domain, indicating where each spectrum used in the purity calculations was obtained. Also shown are the spectra used for background subtraction (if desired).

For methods development, automated library search software allows the chemist to select a variety of criteria on which to perform the search, including peak name, retention time, and match level. The search can be performed either automatically as part of each analysis or done manually post-run.

### Automated structure elucidation based on spectroscopic data

*Reinhard Neudert, Chemical Concepts GmbH, D-69469 Weinheim, Boschstr. 12, Germany*

Interpretation expert in a spectroscopic laboratory are supported by computers, primarily for:

(1) Automating the interpretation process.
(2) Producing unbiased results leading the expert to the correct solution.

SpecInfo is a structure elucidation system, based on libraries containing structures and assigned spectra. Nuclear magnetic resonance, mass spectrometry and infra-red spectroscopy are represented as the most important projections of structural features.

Starting from the spectra of an unknown compound, library searches for the most similiar spectra are performed. The resulting hit lists are analysed for statistically relevant substructures which are introduced into the goodlist-input of the structure generator, together with the molecular formula. The generator produces all possible isomers. To make the interpretation convenient for the expert, an automatic reduction of the solution set is required. Probably the most powerful tool for this is CNMR spectrum prediction; the predicted spectra for the generated structures are matched against the CNMR spectrum of the unknown compound. Only a few structure proposals remain which have to be checked by the expert. Since CNMR spectra are not always available, predictions of IR and MS spectra are desirable. Semiempiric calculations together with neural net approaches can solve this problem.

This work described the procedures outlined above based on practical examples. Inherent difficulties as well as efficient solutions were discussed.

### The use of artificial intelligence in the experimental design and optimization quantitative methods using vibrational spectroscopy data

*M. P. Fuller and R. C. Weiboldt, Nicolet Instrument Corporation, 5225 Verona Road, Madison, WI 53711*

Historically, commercial quantitative analysis software packages for the analysis of vibrational spectroscopy data have relied on the analyst to determine analysis feasibility, develop an appropriate set of standards, choose the best algorithm and optimize the algorithm parameters to produce the final method. The paper discussed the use of a new quantitative analysis software package based on an artificial intelligence approach to quantitative and qualitative method development. In this approach the analyst first enters an interview section where information is entered about the total sample composition, components of interest, physical properties and accuracy and precision expectations. From this information an analysis approach and compositional specifications for several standards is suggested. The spectra from this small set of standards is then used to determine the analysis feasibility and the required algorithm. Next, the complete list of recommended standards is generated, the samples analysed and the method parameters automatically optimized. This approach has been used to develop methods for the analysis of aromatic hydrocarbon mixtures, multi-vitamins and for ambient air monitoring. The development phases for these models were described.

### Application of modified statistical model of overlap to gas chromatograms having variable peak densities

*Matthew E. Johll, Department of Chemistry, University of Wisconsin, Platteville, WI 53818 and Joe M. Davis, Department of Chemistry and Biochemistry, Southern Illinois University at Carbondale, Carbondale, IL 62901-4409*

Statistical theories have been used in the past decade to estimate the likelihood that adjacent single-component peaks (SCPs) overlap in high-resolution separations. The motive for these activities is a comparison of the number of observable peaks to the estimated number of SCPs; this comparison gauges the extent or completeness of separation. One of the simplest of these theories, the statistical model of overlap (SMO), rests on the assumption that the average number of SCPs in any small interval of the separation is identical. This assumption is not satisfied in many practical cases, in which the density of SCPs (i.e. the number of SCPs per interval of separation space) varies throughout the separation.

The SMO has been modified to address the variation of SCP density. This modification was tested and verified by computer-simulated chromatograms and one experimental chromatogram. The major attractive feature of this modification is that one can avoid the time-consuming and frustrating empirical search for experimental separation conditions, under which the classical SMO can be applied. Instead, one can use typical experimental conditions to develop the separation quickly.

The authors reported further experimental verification of this modification by gas chromatography. In brief, chromatograms of a lime-oil mixture were developed very simply at constant heating rates of 2 to 8°C/min (i.e. at heating rates commonly used in research and clinical laboratories), and the number of SCPs in the resultant chromatograms was estimated by modified theory. For these chromatograms, no attempt was made to establish conditions under which the classical SMO was applicable. The same mixture then was chromatographed under conditions applicable to the classical SMO, and the number of SCPs again was estimated by classical SMO theory. The numbers of SCPs so estimated from both series of chromatograms were identical within 10%, once the sensitivity of the detector to very minor peaks was corrected. To test further the modified theory, the first third or so of a series of lime-oil chromatograms was developed at 2°C/min, and the remaining two-thirds was developed at 8°C/min. The heating rates then were reversed to develop the first third of a series of chromatograms at 8°C/min and the remaining two-thirds at 2°C/min. For both combined heating rates, chromatograms of extremely variable density were produced. The interpretation of these chromatograms by the modified theory again provided estimates of component number within 10–15% of the classical SMO. Thus, it appears that the tedious search for experimental conditions prerequisite to the classical SMO can be avoided.

Procedures for the implementation of these estimations were discussed. Only the retention times of the observable maxima are necessary to the estimation, and these are readily determined by commercial integrators.

### Simultaneous, automated determination of nitrogen oxides, aldehydes, and ketones in air

*Andreas H. J. Grömping, Uwe Karst and Karl Cammann, Chair of Analytical Chemistry, Institute for Inorganic Chemistry, Westfälische-Wilhelms-Universität Münster, D-4400 Münster, Germany*

Nitrogen oxides, aldehydes and ketones are significant environmental pollutants and gaseous poisons. Since almost everyone is frequently exposed to them, especially to formaldehyde and/or nitrogen oxides, it is important to know the risks involved. Because both are formed as combustion byproducts, they frequently occur together (for example in automobile exhaust, candle smoke, and tobacco smoke). The authors reported the first method for simultaneous determination.

The method is based on the well-established and practical DNPH-HPLC-method for the determination of aldehydes and ketones. In this method DNPH reacts with the aldehydes and ketones to form the corresponding hydrazones. The reaction of DNPH with nitrogen dioxide to the corresponding azide was used for the determination of nitrogen oxides.

The azide shows a strong absorbance in a similar range so that it can be detected easily. Different methods of sampling were studied. Detection limits are 50 ppb for impingers, 10 ppb for solid sorbents (sampling at 0.8 l/min for 15 min), and 150 ppb for passive sampling (sampling for 4 h).

Furthermore, the development of an automated method for the simultaneous determination of nitrogen oxides, aldehydes and ketones was described. All components are sampled together by means of impingers, which are filled with an acidified solution of DNPH in acetonitrile. The corresponding derivates are separated and quantified by reversed-phase HPLC with UV detection at 300 nm or at 345 nm. Using this reproducible method calibration curves with a correlation coefficient of 0·999 could be obtained. The method was validated by means of several field tests, measuring candle smoke and cigarette smoke.

### Quantitative determinations using an on-line SFE/FTIR interface based on chalcogenide fibre-optics

*Daniel L. Heglund, David C. Tilotta, University of North Dakota, Department of Chemistry, Grand Forks, ND 58202-9024, Steven B. Hawthorne and David J. Miller, Energy and Environmental Research Center, University of North Dakota, Grand Forks, ND 58202*

On-line SFE/IR using high pressure flow cells has recently shown promise as a means of eliminating the collection solvent as well as the need for flow restrictors. To date, on-line SFE/IR interfaces have employed thick, IR transparent windows. These windows are expensive ($200–$400 each), optically poor (70% transmittance), and are not easily cleaned.

Recent work in the authors' laboratory has focused on the development of an on-line interface based on chalcogenide (AsSeTe) fibre-optics. Chalcogenide fibre-optics are inexpensive ($1·50/cm), easily replaced if contaminated, and have excellent optical transmission properties in the 5000–800 $cm^{-1}$ spectral region.

As an application example of the fibre-optic SFE/FTIR interface, total petroleum hydrocarbons (TPH) are determined in soil. Analytical calibration was accomplished by monitoring the C–H stretching region at 2932 $cm^{-1}$. Calibration curves were made from the EPA standard reference oil as stated in method 3560. On-line SFE/FTIR offers low part-per-million (ppm) detection limits and linear dynamic ranges of at least two orders of magnitude ($r = 0{\cdot}990$). Analysis of 'real world' soil samples by on-line SFE/IR gives reasonable agreement with Soxhlet extractions.

### On-line determination of moisture in HCl

*R. T. Talasek and J. D. Hogan, Texas Instruments, Inc., PO Box 655012, M/S 301, Dallas, TX 75265*

Moisture in hydrogen chloride distribution systems used in semiconductor manufacturing is a major destructive force due to resulting corrosion. Perhaps even more significant is the potential wafer yield loss due to additional metallic particles resulting from corrosion. Several laboratory techniques currently exist for the determination of moisture in HCl. However, moisture measurements in the gas distribution system utilizing current techniques are cumbersome, if not impossible. Recently, a technique has been developed that allows measurement of moisture in HCl distribution lines on a real-time basis. A flow cell was constructed of electro-polished stainless steel tubing and other standard components typically used in distribution systems. The path length of the flow cell can be several metres long, and is limited primarily by the length of 'straight-run' tubing in the distribution system. The moisture content of the gas passing through the flow cell is monitored by infra-red spectroscopy, utilizing a fibre optic interface between the cell and the source and monochrometer. Not only does this system allow the background moisture content of the HCl (with system contributions) to be monitored, but also contributions from events such as cylinder change-outs and valve switching can be observed.

### On-line protein analysis using coupled capillaries for immobilization of multiple enzymes and capillary electrophoresis

*Larry J. Licklider and Werner G. Kuhr, Department of Chemistry, University of California, Riverside, CA 92521-0403*

The object of the work reported in this presentation was to produce an effective means of manipulating sample in coupled fused silica capillaries of 50 micron inner diameter. Enzyme immobilization onto the inner surface of one capillary, using biotin–avidin–biotin technology, allows the formation of a variety of chemical microreactors having different specificity towards peptide bonds and the carbohydrate–peptide linkages of glycopeptides. The authors have developed a capillary electrophoresis system for fast on-line peptide mapping and sequence analysis of proteins, including glycoproteins. Generally, there is a requirement to increase accessibility of peptide bonds to the endoproteinase by denaturing the native protein because most native conformation proteins are resistant to proteolysis.

Exposure of the protein to denaturing conditions takes place in the electrophoresis medium inside a capillary microreactor. Transfer of denatured protein into an endoproteinase-modified capillary is accomplished in a fluid junction. Because the electrophoresis medium is of different composition and incubation time must vary in each capillary microreactor, a third capillary shares the fluid junction and serves as an inlet for electrophoresis buffer and the chosen reaction medium. After transferring sample, electrophoretic flow is halted, the denaturation capillary is sealed off, and enzyme hydrolysis occurs for an appropriate time before flow is re-established. It is necessary to switch the electrophoresis fluid flow before transferring digested protein and our design accomplishes this simply, without need of reconfiguration of the capillary junction. Capillary electrophoresis (CE) separation capillaries are available to receive fluid junctions transfers of the material in any capillary microreactor. An important factor to control is the direction and magnitude of electroosmotic flow in each capillary. The experimental design presented is complementary with dynamic modification of the electrophoresis medium to control peptide-wall interactions.

### *In-situ* fermentation analysis with a fibre-optic near-infra-red monitor

*Yue Li, Chi-Shi Chen, Chris W. Brown, Department of Chemistry; James W. McCrady, Fang-Ming Sun and Richard W. Traxler, Department of Food Science and Nutrition; University of Rhode Island, Kingston, RI 02881*

The art of fermenting beverages has grown into a multibillion dollar industry. The rapid and continuous measurement of changes in the concentrations of constituents in fermentation broth is very important. A noninvasive method is desirable because sterility problems can be avoided and sampling processes can be simplified.

Due to the simplicity and speed of Near Infra-red Spectroscopy (NIRS), it is a very promising technique in the fermentation industry. NIRS provides a convenient analytical method for determination of major chemical constituents in a fermentation broth. Coupled with fibre optics, NIRS offers an attractive method for *in situ* fermentation analysis. Major advantages of this technique are that it is nondestructive to the fermentation broth and it can provide real time analysis.

This paper discussed the application of a miniature fibre optic NIRS monitor for the *in-situ* measurement of glucose and ethanol content during glucose fermentation. A bifurcated fibre optic probe is used to transmit the near-infra-red light to and from the fermentor; the transmitted light is reflected back to the fibre bundle by a first surface mirror which is placed 0·8 cm from the end of the fibre. The monitor consists of a miniature holographic grating monochromator with a germanium photodiode array detector. It operates in the 1100 to 1450 nm region. The spectrometer is rugged, compact and reliable; thus, it is ideal for on-line measurements. Results from several different fermentations were presented.

### HPLC determination of sugars and organic acids in food and beverages using a fully automated on-line dialysis technique for complex matrix purification

*Eric Verette, Francois Qian and Fabrice Mangani, Gilson Medical Electronics (France) S.A., 72 rue Gambetta, B.P. 45, 95400 Villiers-le-Bel, France*

In the field of automated sample preparation, the current trend is for simple, quick, robust and economical techniques. However, this goal becomes increasingly difficult to achieve for samples containing complex matrices, such as proteins, high molecular-weight polysaccharides and condensed polyphenolic compounds. In food analysis, where such complex matrices are regularly encountered, the need for more involved sample preparation protocols is common. A protocol may involve several sample preparation steps, for example filtration, centrifugation, extraction, some of which may be difficult to fully automate. An original and totally automated approach to the preparation of samples containing complex matrices, is ASTED. ASTED is based on a unique combination of on-line dialysis, to eliminate microparticles and macromolecules from the liquid sample, with on-line SPE, to pre-concentrate analytes before analysis, generally by HPLC. A wide range of applications have already been developed on ASTED for the analysis of biological matrices in both clinical and pharmaceutical fields. This work demonstrates the suitability of this on-line dialysis technique in the area of food analysis, with particular reference to the determination of sugars and organic acids in several beverages and dairy products.

The study was developed in two steps. First, the chromatographic conditions were optimized according to the analyte of interest. An amino-bonded column was used to determine sugars in dairy products, and an ionic column was used for the simultaneous determination of sugars and organic acids in alcoholic and non-alcoholic beverages; both systems used refractive index detection and calibrations were performed using internal standards. The analysis times for the dairy products and beverages were 15 and 35 min, respectively. The second step was the optimisation of relevant sample preparation parameters. The concentrations of analytes and the nature of the samples did not necessitate the need for pre-concentration; and of the nine dialysis modes possible with ASTED, the pulsed (donor)/static (recipient) mode gave the best recoveries. All sample preparation times were inferior to their corresponding chromatographic times, and thus did not affect the overall analysis times.

This application permitted the determination of six organic acids, four sugars, ethanol and glycerol from liquid food stuffs having complex matrices, with a detection limit of 0·1 g/l and an average coefficient of variation of 3%. These characteristics correspond widely to the criteria required by the food industry for routine analyses. In addition, no organic solvents are used during the ASTED process so the technique addresses present and future environmental requirements.

### Aspects of automated, high-sample-throughput capillary electrophoresis

*David N. Heiger, Martin Verhoef and Mike Thomas, Hewlett–Packard GmbH, Hewlett-Packard Straße, 76337 Waldbronn, Germany*

Capillary electrophoresis (CE) is rapidly becoming a routine technique in separation science. It is often used in conjunction with other techniques, primarily liquid chromatography (LC), since the electrophoretic separation mechanisms yield complementary information. Although CE is a relatively young technique it has grown rapidly, primarily with respect to its application range. As a result, CE users are requiring instrumentation offering a high level of automation, high sample throughput, and reliability associated with the more mature separation techniques such as liquid and gas chromatography.

Instrumental requirements as they relate specifically to CE were discussed. The presentation emphasized automated method development, off-line buffer replenishment, and spectral library searching. In addition, GLP tools comparable to those required for LC, such as system suitability/validation reporting, instrument status log books, and automated baseline noise measurements, were described.

### Monitoring of coal liquefaction processes using fibre optic FT-IR spectrometry

*Anthony S. Bonanno, Michael A. Serio, Stuart Farquharson, David S. Pines, Kim S. Knight and Erik Kroo, Advanced Fuel Research, 87 Church Street, East Hartford, CT 06108.*

The continued development of coal liquefaction processes would be greatly enhanced by the availability of on-line diagnostics that would allow one to 'see' inside the various process stages and monitor such parameters as catalyst activity, upgrading of liquids, depletion of solvent and heteroatom removal. This work described the application of FT-IR diagnostics to the characterization of coal liquefaction process streams. A sapphire optical fibre was used as an attenuated total reflectance (ATR) element to probe the harsh liquefaction process stream. ATR provides a short, reproducible pathlength which allows for the analysis of highly absorbing materials, such as liquid hydrocarbon streams, and the properties of sapphire are well suited for the analysis of high temperature and high pressure process streams. A test cell was constructed which allowed *in situ* monitoring of coal liquefaction reactions at temperatures up to 400°C and pressures up to 3000 psig. A sapphire fibre optic ATR sensing element was coupled to an FT-IR spectrometer using zirconium fluoride fibre optic cables. The ability of the system to follow liquefaction chemistry was demonstrated by conducting laboratory-scale liquefaction experiments with coal samples. The spectra were used to determine liquid composition parameters such as aromatic/aliphatic CH, methyl/methylene CH, and hydroxyl distributions. Correlations were obtained between changes in these parameters and changes in the yield of soluble products obtained by independent measurements.

### Automatic hydrocarbons group type separation for fuel sample characterization by on-line LC-GC

*A. Trisciani, F. Munari, F. Andreolini and A. Sironi, Fisons Instruments, Strada Rivoltana, Rodano, Milan, Italy*

The advantages of on-line coupling of LC to capillary GC over off-line preparation methods has been extensively explained by various authors. These advantages are clear when more than one class of compounds has to be separated in a complex sample. A typical case is the determination of aliphatics, aromatics and other classes of compounds in fuel samples.

This paper described an LC group-type separation followed by the automatic sequential transfer of the several LC fractions to GC for components separation. Two HPLC methods were used to perform the class separation of the fuel samples: column backflush; and precolumn and column set-up backflushing the precolumn.

With the first procedure a sample is injected onto an LC silica column, forward elution allows the separation of the fraction containing the aliphatic hydrocarbon and its transfer to the GC. Then the LC column is back-flushed for the elution of the total aromatic fraction that is successively transferred to GC as a single fraction. Using the second method two columns are used. As before the sample is injected onto an LC silica column which works exactly as in the first case, but then the backflushed aromatic fraction is transferred to a second LC column packed with amino bonded silica. This amino column allows the separation of the aromatics according to the number of rings in the polycyclic aromatic hydrocarbon structure, each corresponding fraction is then automatically and sequentially transferred to the GC for component separation.

Reproducibility data for each hydrocarbon fraction, obtained with both separation methods, were presented.

### Automated simulated distillation using an articulated laboratory robot

*William F. Berry and Vince Giarrocco, Hewlett–Packard Co., 2850 Centerville Road, Wilmington, DE 19808*

There are several ASTM methods for simulated distillation (SIMDIS) of petroleum products. Each method is specific for a class of compounds within a certain boiling range. Although sample preparation for SIMDIS is relatively straightforward, it is time-consuming, monotonous, and subject to errors. Automation is able to address each of these issues as well as matching or improving the precision of manual sample preparation.

SIMDIS sample preparation, analysis, and data handling for ASTM method D2887 is automated using a laboratory robot system and necessary peripherals. The articulated robot is used to transport samples among workstations and deliver the prepared samples to a gas chromatograph. The laboratory operations involved in the automated process are:

(1) Weighing test tubes, sample, and solvent.
(2) Capping and uncapping test tubes.
(3) Dispensing carbon disulphide.
(4) Mixing the sample.
(5) Transferring a sample aliquot to a GC vial.
(6) Crimp capping vials.
(7) Delivering vials to an autosampler tray.

The robot system software controls all devices on the bench and communicates to other software control packages on a single computer. Included in this are the following operations:

(*a*) Create a SIMDIS sequence based on the number of samples prepared.

(*b*) Automatically transfer all sample information (name, weight) to the SIMDIS software.
(*c*) Automatically transfer the weight of carbon disulphide dispensed to the SIMDIS software.
(*d*) Automatically load and start a chromatographic sequence.

Specific details of the automated method, including chromatographic results, were presented. Ideas and plans for future work that would incorporate automated sample prep and analysis for other ASTM methods were discussed.

### Automated liquid/liquid extraction of aqueous environmental samples for pesticides and PCBs

*Kevin P. Kelly, Loren C. Schrier, Evelyn E. Conrad and Nancy L. Schwartz, Laboratory Automation Incorporated, 555 Vandiver Drive, Columbia, MO 65202*

Aqueous environmental samples are commonly analysed for halogenated pesticides and PCBs. The traditional method for segregating target analytes from matrix material is liquid/liquid extraction in separatory funnels or in a glass continuous liquid/liquid extraction apparatus. Much manual labour is required and serious impediments to adequate extraction efficiency, such as emulsion formation, frequently occur.

A newly developed automated liquid/liquid extraction system prepares methylene chloride extracts of up to six samples simultaneously. No shaking is involved; intimate phase mixing is accomplished through application of an electric field to droplets of water phase which are suspended within the non-conducting extraction fluid. All phases of the extraction process are automated, including metering and delivery of extraction solvent, timing of process stages, and rinsing of the extractor at the conclusion of the extraction process.

An extraction set can be completed in three hours or less. The instrument processes aqueous samples traditionally extracted, for example, under US EPA Methods such as SW-846 3510 or 3520. Many instrument operation parameters are selectable, and the system is easily operated by laboratory technicians after setup. Extracts are automatically collected in a conventional (glass) bottle for easy transfer and storage.

Various samples were prepared using the automated extractor. Extracts were evaporated with solvent exchange to hexane and samples were analysed by GC/ECD. Target analyte recovery and reproducibility data were presented for some chlorinated analytes. Principles of operation of the instrument were described.

### The use of on-line ion chromatography for high temperature and high pressure reaction studies

*Garry J. Lynch, Westinghouse Electric Corporation, Bettis Laboratory, PO Box 79, West Mifflin, PA 15122*

This paper described the use of on-line ion chromatography as a tool for chemistry reaction studies in small volume systems. The technique was used to study chemistry behavior in a high temperature and high pressure autoclave system. A duel analyser, multi-channel on-line ion chromatograph (IC) was configured to automate the sampling and analysis of the chemistry in the test. The analytical channels were set up for analysis of inorganic anions, monovalent cations, conductivity, and pH. Conductivity and pH were measured by using the IC as a flow injection analyser.

The use of the IC system provides significant advantages over conventional sampling and analysis techniques. These advantages include:

(1) A significant reduction in sample volume (by about a factor of 10 for this testing) which results in much less impact of sampling on the process being studied.
(2) A closed sampling system that protects air or light sensitive analytes from breakdown in the sampling and analysis process.
(3) Around-the-clock test performance combined with automatic calibration and quality control checking capabilities due to the automated nature of the technique.
(4) Detection and tracking of reaction products or unexpected contaminants.

The method used to correct the measured chemistry concentrations for the effects of sampling and the method used for the calculation of control chemical loss half-lives were presented. A limited evaluation of the flow injection analysis methods for conductivity and pH was also provided.

### Batch synthesis process scale-up: Part I—Composition monitoring by infra-red reflectance spectroscopy

*Debra Younger, James T. Leach and Randell Russell, Development and Control, Arkansas Eastman Division, Eastman Chemical Company, Batesville, AR 72503*

Arkansas Eastman Division of Eastman Chemical Company (ARK) has a multipurpose batch plant which is used for manufacturing a wide variety of specialty chemicals. Technical personnel supporting the batch plant must be able to predict accurately the behaviours of new processes in the ARK batch plant in order to evaluate new business opportunities quickly and efficiently. ARK's ReactIR FTIR spectrometer/reflectance probe system is used to generate near real-time composition/time information, having low random noise levels, to facilitate predictive process modelling.

The ReactIR/reflectance probe system is being used to monitor many types of processess including acetylations, alkylations, sulfonations, chlorinations, and esterifications. Process monitoring is feasible when multiple phases are present in a reaction mixture. Data acquisition and evaluation are accomplished using a Windows-based software package developed by Applied Systems, Inc., personnel. This software facilitates development of simple quantitative FTIR methods while data is being obtained from batch runs, and transformation of composition/time outputs into ASCII tables which are compatible with various kinetic modelling software packages, including SimuSolv.

### Batch synthesis process scale-up: Part II—Use of analytical inputs in development of batch process models

*James T. Leach, Debra Younger and Randell Russell, Arkansas Eastman Division, Eastman Chemical Company, Batesville, AR 72503*

Batch synthesis process scale-up at the Arkansas Eastman site of Eastman Chemical Company is assisted by use of an integrated process development/modelling system which includes a Mettler RC1 reaction calorimeter, a ReactIR FTIR spectrometer/reflectance probe system (Applied Systems, Inc.), and BatchCAD, Ltd.'s REACTION Batch Process Simulation and Modelling software. Analytical inputs, such as composition/time data acquired by methods described in Part I (see preceding abstract) and reaction calorimetry and physical properties data obtained using the RC1, along with plant batch reactor design data and utilities data are used to build plant process models. The models are used to predict allowable plant reagent addition rates and likely temperature profiles. Such information is used to help minimize plant cycle times and to facilitate evaluation of various failure scenarios in process hazards analysis work.

Integrated process development/modeling system technologies provide extensive leveraging of capabilities of individual process development personnel. Further, in the context of economic efficiency, such technologies facilitate very high levels of application of analytical information. By using analytical inputs to build predictive models, optimizations of batch processes, using 'virtual plant reactors', are being performed while processes are still in the laboratory stage; thus, process improvement benefits are realized during their entire plant stage lifetimes rather than after otherwise expensive start-ups and inevitable delays of process improvement work

### Automated optimization of polar mobile phase modifiers in HPLC methods development

*Thomas E. Wheat and John A. MacKay, ATI Instruments North America, The Schrafft Center, 529 Main Street, Boston, MA 02129*

Automated, computerized methods development systems for HPLC have generally focused on optimization of the organic component of the mobile phase. It is, however, also critical to adjust the pH, ionic strength, and ion pairing properties of the aqueous component, particularly when the sample includes ionizable compounds. The Unicam Diamond Solvent Optimization System has been adapted for the systematic investigation of these variables. This system includes a set of software tools for the interpretive optimization of solvent composition. Typically, blends of methanol, acetonitrile, and tetrahydrofuran with a fixed aqueous component are examined. The process can be inverted where an organic mix is selected and held constant while pH, ionic strength, and ion-pair chain length are varied. In practice, an isoelutropic plane is identified across the range of aqueous components. Individual peaks are identified and tracked across this plane using the chemometric techniques of principal component analysis and iterative target transform factor analysis. Retention is modeled using a piece-wise quadratic fit, and the optimal aqueous buffer is identified. This approach was described for samples including common food additives, vitamins, and decongestant/expectorant/antihistamine mixtures.

### Automated determination of UV molar extinction coefficients using an HPLC diode array detector

*Jean-Louis Excoffier and John J. Robinson, Varian Chromatography Systems, 2700 Mitchell Drive, Walnut Creek, CA 94598*

Extinction coefficients are routinely measured using a dedicated standalone spectrophotometer. By using an HPLC Diode Array Detector it is possible to simultaneously determine the extinction coefficient and the purity of the compound analysed.

UV diode array detectors traditionally provide spectra expressed in absorbance units. A quantitative method based on spectral data recently introduced the concept of area spectrum. An area spectrum results from the baseline-corrected integration of a chromatographic peak at each wavelength, and is expressed in units of absorbance multiplied by time. The magnitude of the area spectrum for an elution peak corresponds to the amount injected. The authors demonstrated that by knowing the flow rate and the flowcell's path length, the molar extinction coefficient at each wavelength can easily be derived from the area spectrum. They presented a system in which this computation has been automated and combined with a purity assessment. The precision and accuracy of this determination of the extinction coefficient over a wide range of conditions for pharmaceutical compounds was shown.

### On-line microlc/electrospray ms: A powerful tool in bioanalytical and pharmaceutical applications

*F. Andreolini, A. Trisciani, E. Suardi, C. Borra, Fisons Instruments, Strada Rivoltana, Rodano, Milan and A. Bashall, Fisons Instruments Inc., 55 Cherry Hill Dr., Beverly MA 01915*

Liquid chromatography using microbore (1 mm ID) or packed capillary (0·32 mm ID) columns (microLC) has grown into a reliable routine separation technique capable of dealing with small sample volumes. Low flow rates typically used in microLC (1–100 μl/min) are readily accepted by the Fisons electrospray interface of the mass spectrometer without any flow splitting.

The on-line combination of microLC with electrospray mass spectrometry (microLC/ESMS) allows large, thermally labile, polar or involatile constituents of complex mixtures to be characterized by molecular weight in real time as they are eluted from the microcolumn.

Pharmacokinetic studies require high reproducibility coupled with a sensitive and selective detector. Therefore microLC/ESMS is ideally suited for this kind of study. Other typical applications for this hyphenated technique are the identification of the molecular weight of unknown compounds, from 100 Da up to over 100,000 Da and structure elucidation.

The electrospray interface requires a highly stable and pulseless flow rate, therefore microLC dedicated syringe pumps are the best choice for LC/ESMS. A thorough evaluation of the performance of a dedicated microLC system in terms of reproducibility of retention time and gradient execution at low flow rates will be presented. MicroLC/ESMS analysis for various target compounds in bioanalytical, pharmaceutical and environmental fields was shown.

### Simultaneous determination of chlor-alkali industry products and by-products using flow injection analysis

*Kevein Switala, Lachat Instruments, Inc., 6645 West Mill Road, Milwaukee, WI 53218-1239*

Flow injection analysis methods of chlor-alkali samples must be rugged and precise. This was the objective in the development of the four methods described in this presentation.

A multi-channel instrument was used to determine the concentrations of sodium hydroxide, sodium chlorate, sodium hypochlorite, and sodium chloride in the chlor-alkali samples.

Sodium hydroxide is determined with the development of a colour chelate. The calibration is linear and precision is 0·30% RSD. Sodium chlorate determination depends upon the oxidation of Fe (II) by the chlorate ion. The calibration is linear and the precision is 0·90% RSD. Sodium hypochlorite is converted to chlorine gas in a highly acidic matrix and the chlorine gas diffuses through a Teflon membrane. The chlorine decolourizes the methyl orange indicator which is also in a highly acidic matrix. The calibration is linear, except at very low concentrations, and the precision is 1·3% RSD. The sodium chloride analysis is achieved with the use of a dialysis block and membrane. A linear calibration is obtained for the specified range and the precision is 0·12% RSD. Possible interferences to the determination of the analytes were examined in the paper.

### On-line SFE-GC method for monitoring volatile fuels from soil

*Larry E. Gerdom, Department of Physical Sciences, University of Mobile, PO Box 13220, Mobile, AL 36663 and Howard T. Mayfield, U.S. Air Force Armstrong Laboratory, Environics Directorate, AL/EQC-OL, 139 Barnes Drive, Suite 2, Tyndall AFB, FL 32403-5323*

Previous investigators have reported on the use of supercritical-fluid extraction (SFE) for the analysis of soils contaminated with diesel and less volatile fuels. Off-line SFE with the collection of analytes in a solvent has been the most common SFE technique for use with these compounds. Problems have been encountered in the off-line collection of supercritical fluid extracts of volatile fuels, due to the loss of volatile components. Prior studies have also reported the use of on-line supercritical-fluid extraction with gas chromatography (SFE/GC) methods for the analysis of these materials. The authors investigated the use of on-line SFE/GC in which the extract is transferred directly onto a gas chromatography column and thermally refocused with whole-column trapping. This is accomplished by using a heated transfer line to transfer the SFE analytes directly to the injection port of a GC during the dynamic extraction of a soil sample. The GC column is cooled with liquid nitrogen to $-50°C$ during the extraction. Other operating parameters and instrumentation were described in this presentation. An SFE/GC method for the analysis of JP-4 in a sandy soil matrix was presented. The method has been found to be quantitative for the volatile components of jet fuel at the parts per million level. GC chromatograms for prepared standard samples were presented.

### Automated development of SFE methods for the determination of total petroleum hydrocarbons in soil

*Lori A. Dolata, Dr. Joseph M. Levy, Athos C. Rosselli and Robert M. Ravey, Suprex Corporation, 125 William Pitt Way, Pittsburgh, PA 15238*

Automated supercritical fluid extraction is a powerful sample preparation technique for the determination of total petroleum hydrocarbons (TPHs) in soil. For this application, automated SFE eliminates lengthy sample preparation procedures that are routinely used in the laboratories. SFE shortens the time for preparing samples from hours, or even days, to just minutes with environmentally friendly solvents and this advantage is further enhanced with the ability to prepare large numbers of samples in an unattended and consistent fashion.

This paper discussed the SFE method optimization for total petroleum hydrocarbons in soil and the analytical results will be statistically charted and graphed for discussion. For this particular study an automated SFE unit was used equipped with a VariFlow restrictor. To represent a real world scenario various soil matrices were extracted and the conditions were varied (i.e. change in extraction pressure and temperature, collection temperature, addition of a modifier, absorbents, sample size and trap packings). Each soil matrix was extracted in replicates using a specific method and the extracts were analysed by EPA method 418.1 (spectrophotometric, infra-red) using a calibration curve that satisfied all EPA QA/QC acceptance criteria. Of particular importance is the ability to use SFE for wet soil samples. Normally, such samples are characteristically plagued with mechanical restrictor plugging problems and overall poor SFE recoveries. Results were presented from experiments that have demonstrated consistent variable restrictor operation for the SFE of soils ranging in moisture content for 10 to > 50%.

### Automating the supercritical fluid extraction, derivatization, clean-up and analysis of chlorophenols from soils

*Charles R. Knipe, Hewlett–Packard, Little Falls Site, 2850 Centerville Rd, Wilmington, DE 19808 Hing-Biu Lee and Thomas E. Peart, National Water Research Institute, PO Box 5050, Burlington, Ontario L7R4A6, Canada*

Supercritical fluid extraction (SFE) is a promising sample preparation technique for handling many of today's solid samples in the environmental laboratory. There are several classes of compounds of interest that are amenable to SFE, such as chlorophenols and polychlorinated-biphenyls (PCBs). One of the most successful methods for the extraction and analysis of chlorophenols has been an *in-situ* derivatization method during supercritical fluid extraction as described earlier. Once derivatized the chlorophenols can easily be detected and quantitated by GC/ECD at very low levels (ppb). Currently this method requires a manual clean-up between the derivatization/ extraction step and the GC/ECD analysis. Another difficult analyte/matrix is the quantitative extraction of PCB'S from fish tissue. PCB extraction is again quite amenable to supercritical fluid extraction with the addition of some minor clean-up steps.

This presentation described the SFE extraction conditions along with the sample handling and extract clean-up considerations. In addition to the extraction procedure, automation alternatives for handling the post extraction clean-up steps were presented. Discussion included operation of the hardware/software system, along with validation and robustness of the automation alternatives.

### Automation of supercritical fluid extraction of herbicides from environmental samples using a laboratory robotics system

*Leetta J. Jarvis and William C. Koskinen, University of Minnesota, Soil Science Dept., 1991 Upper Burford Circle, St. Paul, MN 55108*

Supercritical fluid extraction (SFE) is being increasingly used for extraction of organic chemicals from different matrices. SFE does not use expensive and hazardous organic chemicals, therefore it does not create a waste disposal problem. SFE is also less labour intensive than traditional extraction procedures and the extraction time per sample is less. However, in most instrumentation, the sample needs to be manually loaded and unloaded preventing samples from being run unattended. Our objective was to automate SFE using a commercially available laboratory robotics system to combine the advantage of SFE with the advantages of automation.

A Zymark Zymate II robotics system was used to automate a Hewlett–Packard 7680A supercritical fluid extractor. The robotics system and the SFE were modified and integrated to automate the extraction of herbicides from different matrices and the trapping of different analytes on a solid sorbent and in an organic solvent simultaneously. The robotics system loads and unloads the sample from the SFE instrument, the vial containing analyte trapped on a solid sorbent, and a tube containing analyte trapped in organic solvent. It can also perform the sample preparation necessary for final analysis such as adding internal standards. The SFE instrument error light is monitored by the robotics system to detect voltage change when there is an error in the SFE. The robotic system also monitors the voltage at the SFE instrument chamber switch. The voltage change indicates when the sample is finished being extracted and the robot can open the chamber door and remove the sample thimble.

When using a method with a 33 minute run, the authors were able to increase the number of samples extracted from 14 samples per 8 h day manually to 43 samples per day unattended. The effort to automate SFE using an available robotics system was a success.

### Fully automated air canister analysis (EPA TO-14) by ion trap technology

*William C. Schnute, Jeffrey M. McMillan and Sharon Reiss, Finnigan MAT, San Jose, CA 95134 and Grasby Nutech-RTL 4022 Stirrup Creek Drive, Durham, NC 27703*

Discussion of the ways ion trap technology can be applied to the EPA TO-14 method to meet the needs of the US Clean Air Act Amendments (CAAA) of 1990. By the use of SUMMA canisters, samples taken from various industrial, urban, and rural sites are analysed by ion trap mass spectrometry. The process is fully automated using microprocessor controlled autosampler and cryotrap/ cryofocusing, and is designed for minimal operator intervention once the process is started. Due to the sophisticated nature of the analysis, full QA/QC processing monitors all aspects of the process, generating reports for validation and review.

Examples of standard and samples were presented, showing ultra trace level sensitivity, the effects of cryofocusing, and dynamic range possible using these techniques.

### Large volume sample injection for trace analysis with automatic capillary GC and GC/MS

*Fl Munari, P. Colombo, S. Trestianu, Fisons Instruments S.p.A, Str. Rivoltana, Rodano (MI) Italy 20090 and F. Glisson, Fisons Instruments, Inc., 55 Cherry Hill Dr., Beverly, MA 01915*

The capabilities of capillary GC and GC/MS techniques for trace analysis can be enhanced through large volume sample injection. By injecting hundreds instead of few microlitres into the column the detection limits may be improved by two orders of magnitude.

Special technical solutions were employed for solvent venting and sample transfer into the column. They are based on the experience acquired along the last decade in automatic large volume injections for HRGC and in on line ccoupling of HPLC with HRGC.

These solutions were implemented on GCs and benchtop GC/MS instruments provided with an autosampler able to operate on any injection system with conventional or macro sample amounts.

They permit full automation, quantitative analysis, good reproducibilities of peak areas and retention times, no lost in system separation power and full compatibility with GC and MS detectors.

The instruments peformances for large volume injections were tested for a variety of samples by using cold on column and cold splitless (PTV) injectors. The cold splitless injector can be easily derived from the cold on column by attaching to it a vaporizing chamber.

### Method development and automated sample extraction by SFE

*Les Myer, Jess Damian, Joe Algaier and Joe Tehrani Isco Inc., 4700 Superior, Lincoln, NE 68504*

Although supercritical fluid extraction (SFE) has proven to be an efficient method for extracting various compounds ranging from aflatoxins to waxes, there are major obstacles to the wider adoption of SFE. The first obstacle is the time required for method development. The development of optimized methods, especially when considering the many variables of SFE such as pressure, temperature, flow rate, and modifier concentration, presents the researcher with a myriad of conditions to evaluate. Indeed, any chemist knows that method development can be the most costly and frustrating aspect of research. The solution, of course, is automation. The second obstacle to SFE is the transferring instrumentation and methods from laboratories into a routine analysis or quality assurance environment. This transfer requires a well-proven and robust extraction method that is thorough, painstakingly documented, precise, and straight forward. Again, the solution must be automation.

Using the Isco Model SFX 220, a dual-sample SFE system with automated valves, and the Isco Model SFX 3560, a fully automated 24-sample SFE instrument, the work presented here examined the extraction profile of fat soluble vitamins, from pharmaceutical preparations. Extraction kinetics demonstrating the basic principles of SFE were profiled. The authors presented the need for automation and demonstrated its efficiency in method development and routine sample extraction.

### On-line sample pretreatment system for the determination of cations and anions in concentrated acids and bases

*A. Siriraks, S. B. Rabin and J. M. Riviello, Dionex Corporation, 1228 Titan Way, Sunnyvale, CA 94088-3603*

The determination of trace anions and cations in concentrated bases and acids has been limited by matrix concentration in these samples. In order to analyse trace anions in concentrated bases or trace cations in concentrated acids, the sample is usually diluted to the level at which the sample matrix does not interfere with the analytical measurement. Another commonly used sample pretreatment method is neutralization using ion-exchange resin to remove the interfering matrix ion. This technique suffers from limited ion-exchange capacity and significant blank contribution of the resin, thus compromising detection limits for analytes of interest.

This paper described the construction and use of an electrolytic membrane neutralization device for trace anion determination in concentrated bases and trace cation determination in concentrated acids. These devices serve to neutralize and remove interfering matrix ions. The sample pretreatment design and development, integration with the analytical system and analytical data were presented.

### Automated SFE with on-line spectroscopic analysis

*Phillip B. Liescheski, Dale L. Clay, Joe Tehrani, Isco Inc., 4700 Superior, Lincoln NE 68504, Paul D. Seemuth, DuPont Fibers, Kinston, NC 28502-0800 and Terry O. Trask, DuPont Fibers, Waynsboro, VA 22980*

Supercritical Fluid Extraction (SFE) using inexpensive and inert $CO_2$ continues to hold promise in replacing traditional solvent extraction, especially those based on Freon or toxic, flammable solvents. When the advent of automated SFE, the sample preparation cost and time have been reduced; however, the extracts still need to be introduced to the analytical assay device. Several different approaches have been made to address this problem.

One approach is on-line infra-red analysis coupled to automated SFE (i.e. SFE-IR). In this approach the extractant fluid stream flowing from the sample matrix is transferred through a temperature-controlled line to a high-pressure IR flow cell. The fluid pressure within the IR flow cell is maintained by some flow restriction device downstream. An IR spectrometer is used to continuously monitor specific analytes in the extractant fluid stream. By tuning into a specific wavelength band, one can monitor specific analytes or classes of analytes, for example the $v_2$ band for water near 1600 $cm^{-1}$ or the C-H stretch band near 3000 $cm^{-1}$. This technique can be easily coupled also to on-line ultraviolet analysis (SFE-UV) which could be used to differentiate between aliphatic and aromatic hydrocarbons.

Applications suitable for this approach are:

(1) Quality assurance analysis of finishing oils on textile fibres.
(2) Environmental Total Petroleum Hydrocarbon (TPH) analysis of soil.
(3) Water content analysis of dry goods.

In this presentation the SFE on-line spectroscopic approach and related applications were discussed along with experimental results.

### Development of a standard operating procedure for the continuous emission monitoring or incinerator effluents using FTIR technology

*Elizabeth Y. Hwang, Jack C. Demirgian, Analytical Chemistry Laboratory, Chemical Technology Division, Argonne National Laboratory, 9700 South Cass Avenue, Argonne, IL 60439, Zhuoxiong Mao, Energy Systems Division, Argonne National Laboratory, 9700 South Cass Avenue, Argonne, IL 60439 and Grant Plummer, Entropy, Incorporated, 8724 Glenwood Avenue, Raleigh, NC 27612*

Disposal of hazardous wastes by incineration is the preferred technology from an economic standpoint. However, lack of convincing data to demonstrate the safety of incinerators has led to public concern regarding potential health hazards resulting from the emission of toxic organic compounds into the atmosphere. Research is being carried out at Argonne National Laboratory (ANL) to develop a mobile Fourier transform infra-red (FTIR) spectrometer for continuous emission monitoring of stack gases. Because incineration is controversial amongst the public, data collected on the FTIR must not only be scientifically valid, but also legally defensible. A protocol for applying FTIR in emission testing has recently been prepared by Entropy, Inc. (Raleigh, North Carolina) under the auspices of the Environmental Protection Agency (EPA). The document provides: (1) general guidelines; (2) techniques for developing and evaluating the methods; and (3) requirements for reporting and quality assurance procedures.

The purpose of this presentation was to describe the recent development of a Standard Operating Procedure (SOP) by The ANL Analytical Chemistry Laboratory (ACL) for continuous emission monitoring of incinerator effluents. This SOP is based on the proposed EPA protocol. It applies to the determination of compound-specific concentrations in multiple-component gas phase samples using mid-infra-red absorption spectroscopy. This SOP was used at a field test conducted at the OAK Ridge Gaseous Diffusion Plant (ORGDP) in August and September of 1993.

This discussion covered the following topics of practical interest: evaluations of analytical regions and software, demonstration of instrument stability and data reproducibility, and validation of training set and continued calibrations. Emphasis was placed on (1) acquisition of robust training set and (2) the use of performance evaluation standards and calibration transfer standards to test system performance and data validity.

### Continuous emission monitoring of incinerators using extractive and passive-remote Fourier transform infra-red spectroscopy

*Jack C. Demigian, Cheryl Hammer and Elizabeth Y. Hwang, Analytical Chemistry Laboratory, Chemical Technology Division, Argonne National Laboratory, 9700 South Cass Avenue, Argonne, IL 60439*

The US Clean Air Act of 1990 requires the monitoring of air toxics including those from incinerator emissions. Continuous emission monitors (CEMs) can address public concern about emissions of hazardous organic compounds by demonstrating the safety of incinerators. Fourier transform infra-red (FTIR) spectroscopy can provide the technology for continuous emission monitoring of stacks. Stack effluent can be extracted and analysed in less than one minute with conventional FTIR spectrometers. Passive-remote FTIR spectrometers can detect certain emission gases over 1 km away from a stack.

The authors discussed advances in both extractive and passive-remote FTIR technology. Standard operating procedures for these systems are being developed and tested. Extractive systems are being tested with EPA protocols, which will soon replace periodic testing methods. Passive-remote FTIR spectrometers have the advantage of not requiring an extracted sample, but they have less sensitivity. The authors have evaluated the ability of commercially available systems to detect fugitive plumes and to monitor carbon monoxide at a coal-fired power plant.

### On-line *in-situ* determination of residual carbon in fly ash

*Anthony S. Bonanno, Peter R. Solomon and Michael A. Serio, Advanced Fuel Research, 87 Church Street, East Hartford, CT 06108*

An instrument capable of making on-line, *in situ* measurements of residual carbon content in fly ash would be a valuable tool for optimizing power plant efficiency and achieving pollution control goals. Current instrumentation is based on extractive point sampling, and is relatively slow, prone to inaccuracies, and requires significant maintenance and sample handling equipment. Instrumentation and methodology based on infra-red emission and transmission spectrometry has been developed for making on-line, *in situ* measurements of carbon in fly ash. Combined FT-IR emission and transmission spectrometry were used to determine the spectral emittance of the fly ash particles, and a correlation was developed between spectral emittance and residual carbon content. Thus, the residual carbon content in fly ash can be determined on a time scale of a few seconds without the maintenance problems associated with an extractive system. Results were presented from shakedown tests in a laboratory scale combustor and from a field test at a pilot scale combustion facility.

### Flow injection analysis of trace levels of aluminium in surface water with micellar enhanced fluorometry

*Zhengyi Zhou and Philip B. Oldham, Department of Chemistry, Drawer CH, Mississippi State University, Mississippi State, MS 39762*

The mobilization of toxic $Al^{3+}$ in natural water systems due to acid rain is a serious problem in the heavily industrialized areas. Aluminium, in the ionic state, has been shown toxic to plants and fish as well as human beings. Current analytical methodologies for monitoring environmental aluminium provide limited information concerning speciation. Therefore, a better understanding of aluminium–water chemistry and the concomitant development of improved methods of speciation monitoring are the primary objectives of this work.

In this paper, the authors presented the use of flow injection analysis for monitoring trace levels of aluminium in surface water based on enhanced fluorometry. Morin is used as a chelating agent reacting with $Al^{3+}$ to form a fluorescent complex in acidic solution. Triton X-100 is used as a fluorescence-enhancement reagent. Compared to the Al-Morin-Cetyltrimethylammonium bromide system, this method has the advantages of a high micelle enhancement factor (MEF), low background, and diminution of interferences. In addition, possible mechanisms and optimal conditions (pH, reaction time, and concentration of salt) for micellar enhanced fluorescence of the Al–Morin complex were discussed.

### The use of automated SFE/GC for the quantitative determination of pesticides in soils, feeds and foods

*Joseph M. Levy, Lori A. Dolata, Robert M. Ravey, Kathy Holowczak and Glenn Muzyk, Suprex Corporation, 125 William Pitt Way, Pittsburgh, PA 15238*

Supercritical Fluid Extraction (SFE) has evolved to a point where it has gained a significant amount of attention by the scientific community. However, widespread acceptance of SFE cannot be attained unless pertinent applicatons can be demonstrated. Such applications were presented and discussed with respect to the quantitative determination of various carbamate, organochlorine, and organophosphorous pesticides in selected soil, feed and food matrices utilizing off-line SFE/GC-MS and ECD. Results were presented from the variation of DFE variables (pressure, temperature, duration and flow) to achieve the highest extraction efficiencies. In addition, modifier augmented SFE was discussed with respect to the extraction of the more polar pesticides.

### Creating and managing distributed lab operations

*James N. Bower and John J. Fitzgerald, Automated Compliance Systems, Inc., 245 Route 22 West, Bridgewater, NJ 08807*

The technology of relational databases, local area networks (LANs), wide area networks (WANs), inexpensive communications hardware, and management software tools make this an ideal time to implement systems to manage distributed lab operations. It is possible to manage geographically distributed systems in ways which historically were not available within a single lab building. This presentation described how distributed lab management systems can be constructed, with attention to how the underlying technology facilitates the needed operational functionality.

Distributed lab operations are found increasingly in both the corporate and contract worlds. Distributed operations may mean anything from simply siting instruments away from the primary lab to completely separate labs managed as a single entity. Physical separation makes it more difficult to capture data, reconcile methodologies, exchange information, and manage labs within acceptable parameters. These problems may in turn become limiting factors for lab growth, acceptability of services, and financial performance in an increasingly difficult climate.

The system described in this work is composed of

(1) The Oracle relational database management system.
(2) Ethernet local area networks.
(3) TCP/IP based wide area networking.
(4) Dial up routers.
(5) An advanced messaging system.
(6) User configurable data capture software.
(7) A graphical user interface based management software tool.

These elements are combined to create a scalable system which can adapt to each of the distributed lab challenges above, and can provide information to management on a timely basis about any aspect of lab operations.

### The development of a LIMS for a multi-functional laboratory

*R. Joseph Lilly, Department of General Services, Commonwealth of Virginia, 805 E. Broad St, Suite B-3, Richmond, VA 23219*

As an alternative to purchasing a LIMS system for a multi-functional laboratory can be effectively developed in-house.

The Division of Consolidated Laboratory Services is the State laboratory for the Commonwealth of Virginia performing environmental, product quality control, clinical and health screening and testing in support of the Departments of Health, Agriculture and Environmental Quality. In existence for over 20 years, DCLS did not have a LIMS when the decision to develop one was made in 1991. Previous efforts to procure a LIMS had not been successful due to the cost involved and the number of modifications necessary to support such a multi-functional laboratory.

This paper discussed the information engineering, rapid application development, integrated CASE tools and code generation methods and techniques that were used in successfully creating a modern LIMS that has a client/server architecture with a relational database engine and is fully portable to many different platforms.

### Implementation and benefits of a LIMS in a research environment

*Gérard Iafrate, Philippe Cleon, Rhône Poulenc Industrialisation, B.P. 166 24, avenue Jean Jaurès, 69151 Decines Charpieu Cedex, France and Claude Gonzalez, LIMS Consultant, 200, rue de la Roquette 75011, Paris*

The difficulties of managing information in a research environment lie in the basis of industrial research:

(1) Most studies are unplanned and changeable, based on all kind of questions. Thus, it is very difficult to implement fully automated procedures, particularly for reporting.
(2) Adapting or developing methods for a specific purpose is the main aspect of the job rather than the number of results that are generated.
(3) Research data may be used several years later for reporting or resumption of research projects. However, searching for past information is often a difficult puzzle.

Therefore, an important potential benefit of information management systems in a research laboratory is the ability to record and track all relevant information related to the results.

Analytical Laboratories of Rhône Poulenc Industrialization, located near Lyons (France) cover all kinds of industrial problems, from process development to environmental studies, using all kinds of analytical techniques. Thus, they are representative of industrial research environment.

To cover the requirements described above, two main functions were developed and integrated into a commercial LIMS.

(*a*) Using a semi-automated reporting interface. The user can build and automate any kind of report into a text editor or spreadsheet using database information.
(*b*) The user can record and track all the steps involved in the development or adaptation of methods by simply entering or modifying sets of operating conditions and comments related to an analytical study.

Commitment of users to the project shows that there is a real need for durability of research information and consolidation of research projects over time in the interest of efficient research work.

Though a LIMS does not cover all the requested functions, it can be the core of a research laboratory information management system. Extension to other scientific software is required for successful implementation.

### The value of prototyping laboratory automation systems

*Cheryl J. Hawley, Laboratory Expertise Center, Digital Equipment Corporation, 601 Parkaire Crossing, Marietta, GA 30068*

Why do so many LIMS implementations fail? Often the root cause is one of communication: asking the right questions, and properly interpreting the results. This applies to requirements specifications, as well as design documents and systems development activities.

A system is frequently designed, implemented, documented, and thrown over the wall to the user community before truly useful feedback is generated. Unfortunately, the feedback is rarely a compliment to the powers of prognostication of the developers, but more likely, vitriolic criticism of the distance between what the users wanted versus what the designers gave them.

This paper addressed the value, logic and planning of effective systems prototyping. Of paramount importance was the inability of even the most methodical systems architects to completely articulate a LIMS environment 'on paper' that will result in the reduction, not the compounding, of bench level work. The resolution of this dilemma resides in the careful development of a prototype system that targeted users can interact with before the final system is cast in stone. The resultant 'real life' feedback is much easier to quantify and qualify, and the final roll out of the system is closely aligned with the user's needs.

As in any major implementation, it is the exceptions to the routine processes that are typically not discovered until the lab personnel start using a LIMS system in their daily functions. By allowing adjustments to the LIMS design while the system is being developed, the prototype in essence validates the LIMS requirements and preliminary design specifications before roll-out, thus lowering the overall system implementation costs by reducing implementation time. It also provides a tested working model that can be scaled up to a fully functional LIMS implementation.

Several key items must be firmly in place to have a successful prototype:

(1) A prototype specification must be written, describing the features that will be included in the prototype. These should include any critical key features of the final system as well as any essential reports needed to utilize the pilot.
(2) Creation of a detailed project plan to ensure time will be allocated to not only the designers, but to the lab participants as well.
(3) Identification of the lab sponsor(s) as well as all lab participants of the prototype. The selection of target labs and personnel to be trained to use the pilot system will directly affect the success of a prototype.

### The development of a Windows based automatic sample scheduler

*M. P. Kelly and G. F. Gostecnik, Analytical Automation Specialists, Inc., 11723 Sunbelt Court, Baton Rouge, LA 70809*

Repetitive sample login has been a tedious process for many PC based Laboratory Information Management Systems (LIMS) in the past. This was particularly true as networks of microcomputers began to replace minicomputers in laboratories where sample loads ranged from several hundred to one thousand samples per day or more.

This paper reported on the development of a full featured automatic sample scheduler to address this need. WINDOWS was chosen as the operating environment, not only for the obvious reasons of popularity and ease of use, but to take advantage of the memory resouces available, multitasking capabilities and interprogram communications using Dynamic Data Exchange (DDE).

The scheduler employs digitized speech in the form of WINDOWS WAV files allowing the user to record appropriate messages for broadcast at sample login. It utilizes features such as a prebuilt pop-up calendar, which not only reduce the program development requirements, but allow the user to modify the program's output using the 'point-and-click' (mouse) interface with no programming knowledge.

When combined with a modern PC based LIMS such as LABWORKS, this system provides a tremendous increase in laboratory productivity by essentially eliminating the need for interactive sample login.

### Using visual basic to provide custom calculations within a chromatography data system

*Larry L. Robison, Soheil Saadat and Vikram Patil, Scientific Software, Inc., 2551 San Ramon Valley Blvd, Suite 205, San Ramon, CA 94583*

The popular Microsoft Windows operating environment not only provides improved ease of use over menubased operatng systems, but makes it easy to exchange data between different software applications. The new Windows for Workgroups encourages the exchange of data between computers in the same network. These software aids combined with today's personal computer power, speed and relatively low prices bring capabilities to today's chemists that were available only to labs with mini computers two years ago.

The EZChrom Chromatography Data System is an example of today's Windows-based applications that provide capabilities required by knowledgeable chromatographers. EZChrom can take data from as many as four chromatographs, each of which may have up to four detectors. Standard report capability in EZChrom provides the user with the ability to format pages with virtually any combination of chromatograms and results text. The user can select from any or all of 27 different results parameters, from retention times to theoretical plates.

If the standard results parameters do not provide the answers the laboratory needs in its reports, five user-definable 'custom parameters' are available for inclusion in reports. Custom parameters calculations can be defined in a Windows executable program written in an easy to learn language like Visual Basic using generic programs provided by the manufacturer as a guide. Custom parameters programs have access to the stored raw data file parameters such as peak heights, retention times, calculated results, etc. The set-up of these calculations provides the option of applying the calculation to individual peaks or to the whole chromatogram. Thus, simple calculations such as area/height ratios or more complex calculations, such as BTU content of a sample, can be made to be a part of every analysis.

If additional data manipulation, such as statistical operations or graphical presentation of many sets of results is required, results can be imported into spreadsheet or database. Export files can be created for individual chromatograms or results can be appended to a file consisting of multiple runs. If completely automatic operation is required, Windows Dynamic Data Exchange can be used to share data between applications. Automatic operation is accomplished by a macro written in the applications macro language to perform the desired tasks.

Examples of various calculations and macros were shown to demonstrate the usefulness of this approach.

### Intelligent linked portability for measuring instruments

*X. F. Gonzalez and R. J. Szymansky, Micro Design Services, Andover, NJ 07821*

A new class of portable instrumentation that combines the measurement instrument with commercially available untethered hand-held computers with wireless communications has been developed for the environmental measurements. By linking the measuring instrument to a hand-held computer with integrated communication, the two independent systems create a flexible intelligent linked measurement instrument.

The hand-held computer is equipped with a Liquid Crystal Display and Touch Screen and supports a series of options including an integrated Spread Spectrum Radio Frequency Transceiver to provide a wireless Local Area Network, integrated Infra-red Transceiver to allow very short distant point to point communications, integrated Laser Bar Code Reader, and signature capture via the Touch Screen.

Portable FID and GC Instruments are to be available with an infra-red link to enable untethered point to point information exchange from the instrument to the hand-held computer.

Facility or outdoor coverage can be implemented via the Spread Spectrum RF wireless LAN allows a collection of hand-held computers to communicate to a PC server via an antenna. In real time, the PC server can be queried by the instrument operators via the hand-held computer regarding the schedule of measurements, type of component to be measured and expected ranges of the values. Measurements with the portable FID or GC can be made by the operator and immediately sent to the PC Base Station for mass storage and evaluation.

Location of components to be measured for gas leaks are confirmed by reading a bar code label attached near the component.

In instances when certification of the measurement is critical, the signature of the individual making the measurement can be captured via signature capture and stored in the master records.

This paper described:

(1) The software and system integration necessary to combine the portable FID and GC Instruments, the hand-held computer, antenna and PC server needed for portable environmental measurements.

(2) The functional features of the overall system including the interface between the portable instruments and the hand-held computer.

This paper provided descriptions of untethered hand-held technology, including wireless real time collection of data, certifications of measurement, delivery of feedback to the operator to aid in decisions, and management tools to improved productivity.

### Analytically precise software selection

*Randy C. Hice, Digital Equipment Corporation, 435 North Eagles Bluff, Alpharetta, LA 30202*

In the past the major challenges to the automation of a sophisticated laboratory operation were finding an affordable computer platform with enough power to serve the need, and searching high and low for a few threads of off-the-shelf software to augment the mountains of custom-written code.

The environment has now changed; the ceaseless shrinkage of staffs and budgets is exacerabated by a rapid increase in competition, impossible project time lines, and a deluge of exceedingly complex vendor software systems which, in an attempt to meet the needs of a diverse market, often come laden with an overkill of functionality. Couple these pressures with a pronounced lack of management compassion for expensive mistakes in time and money, and the phenomenon of 'analysis paralysis' creeps in. The times have also past when a 'no decision' was the safe decision. Top management fully expects remaining middle management to be accountable for the design and implmentation of their strategies. There is to be no more 'throwing over the transom' of concepts and hoping that they will magically germinate into working architectures. 'Darwinian management' dictates that surviving middle managers are renaissance men and women who are scientists, visionaries, business consultants, accountants, and computer technology experts all at once.

How then, does one apply a logical process to software systems selection and development? The answer lies in the matching of business goals and objectives to candidate software systems. This approach seems deceptively obvious at first glance, but, in practice, is exceedingly complex to execute for most organizations. In the spirit of Business Process Improvement, canvassing the user base for 'wish lists' is not a viable methodology for developing justifiable, and defensible system requirements.

This presentation outlined a methodology that smoothly moves through the continuum of the prioritization, and then translation of identified business goals, into terms easily measured against the technical features of baffling software systems. The final software selection is based on the logical mapping of only those features which serve to meet business needs, and discards the window dressing, resulting in a software architecture built on logic, with reduced implementation time, less retro-fitting, and better financial justification.

### Automation of commercial environmental ELISAs for high throughput screening of environmental and agricultural samples

*Christ Shumate, Jim Johnson, Kirk Andree, Hamilton Company, Reno, NV 89502 and Chatan Charan, Midwest Research Institute, Mountain View, CA 94043*

Enzyme-linked immunosorbent assays (ELISAs) for pesticides and herbicides in environmental and agricultural samples are quickly showing their importance in screening applications. Traditional chromatographic methods are expensive and require extended periods of time for results, making them incompatible with on-sight decision making. Often sampling frequency is limited by the time and cost of these methods themselves. ELISAs offer rapid, low cost analysis thereby increasing the frequency of sampling and enhancing the data quality.

Automated ELISA workstations allow the full benefit of these kits to be realized. Sample preparation, reagent pipetting, incubation, washing, and even photometric evaluation can be performed without user intervention. Reliability is increased through the elimination of operator error, better accuracy and precision, and often higher speed. Much larger batch sizes are obtainable and these systems can provide sample tracking with report generation for documentation requirements.

The Ohmicron RaPID assay for atrizine has been automated with the Hamilton Microlab 2200. This is a magnetic particle based assay performed in test tubes. Modifications to the instrument include a pneumatic rack system and dual lumen probe with vacuum capabilities for tube washing.

The Quantix plate based assays for alachlor, metalochlor, and PAHs were pipetted on the Hamilton Microlab AT plus. Soil extracts in methanol were automatically diluated and pipetted into both alachlor and metalochlor test wells contained in the same plate frame. Several dilutions can be programmed to ensure that results fall within calibration range.

The Millipore Envirogard assay for triazines was pipetted on the Microlab AT plus and transferred to the Hamilton Microlab FAME for incubation, reagent addition, washing, and photometric evaluation.

Statistical performance was presented in light of manual procedures and insight into the critical aspects of automating these ELISA kits was discussed.

### The process analytics laboratory in the plant: fit, function and future

*Peter van Vuuren, Exxon Chemical Company, Basic Chemicals Technology, PO Box 4900, Baytown, TX 77522*

In the petrochemical and chemical industries across the world, many Plant Laboratories operate today as stand-alone entities or organizations charged with the traditional responsibility for:

(1) Routine analyses.
(2) Issuing of Certificates of Analysis (CofAs).

(3) Checking the 'correctness' of the on-line analysers on a routine basis or when anybody questions the on-line analyser measurements
(4) Providing calibration standard support to on-line process analysers.
(5) Performing analyses in support of diagnosing troublesome processes.

A case was made in this presentation that this traditional approach is at odds with the Total Quality Management programs being adopted throughout these industries, especially in the areas of inconsistent responsibilities and accountability for plant-wide analytical measurements and wasteful activities, i.e. duplication of on-line and off-line analyses.

The paper introduced the notion that in the context of process analytics as a manufacturing enabling technology, a subset of the traditional laboratory functions is still appropriate but that it should be 'fitted' into and integrated with a *single* organizational function, i.e. 'process analytics'. The charter for the new, streamlined process analytics organization was summarized in terms of continuous improvement, validation support, assistance with technology transfer programs and troubleshooting capabilities. Finally, the primary benefits of the future process analytics organization were highlighted.

### Quality assurance and the process analyser system output

*Linda M. Lane, ARCO Products Company, Los Angeles Refinery, PO Box 6210, Carson, CA 90749-6210*

Use of process analyser systems in the petroleum refining industry is becoming commonplace. These analyser systems are generally designed and installed to operate with minimal maintenance. Their outputs can provide analytical information such as physical, chemical, and product quality properties. This information is typically used for process monitoring, but can also be used for process control, optimization and certification. In order to expand the use of analyser systems for routine control of processes, for the optimization of unit operation and to certify products, well-thought-out controls, tests and inspection activities must be in place.

Such an approach to quality assurance for process analyser systems is achieved through the integration of several refinery departments, including engineering, analytical, maintenance, refinery laboratory, safety and plant operations. The 'ownership' of the quality assurance program may reside in any department, but the neeed for confidence in the process analyser system and its output is critical for successful online applications.

This paper included explanations of process analyser systems presently used in the petroleum refining industry and their relationship to quality assurance. Data from various process analyser systems used for monitoring, optimization and product specifications were also presented.

### Process analytical chemistry: A pharmaceutical perspective

*Paul K. Aldridge and Timothy Norris, Pfizer Central Research, Eastern Point Road, Groton, CT 06340*

Process Analytical Chemistry (PAC), as a relatively new subdiscipline, is finding an increasing number of applications in the pharmaceutical industry. Applying PAC methods in a regulated environment adds additional challenges beyond the traditional technological barriers. After a brief discussion of regulatory challenges, two pharmaceutical applications of Near Infra-red Spectroscopy were described: *in situ* monitoring of a heterogeneous reaction, and the monitoring of the blending process of a model dry powder formulation. In both cases, appropriate chemometrics have been applied for interpretation of the spectral data. It is hoped that applications of this type will eventually lead to parametric release.

### Analytical and process analytical laboratories—Partnership for a successful program in process analyser implementation

*Satish M. Mehta, Paul J. Brimmer, Dennis S. Corrigan, Charles N. Kettler and Michael J. Pearce, Eastman Chemical Company, PO Box 1972, Kingsport, TN 37662-5150*

Since the early 1980s process, analytical chemistry has been described as an 'emerging' subdiscipline of analytical chemistry that is and has been in its most exciting and opportunistic phase. While process analytical chemistry has been fairly successful in the petrochemical industry since the 1950s, its on-site and widespread acceptance in other chemical and polymer manufacturing industries started only recently.

Since 1983, Eastman Chemical Company (ECC) in Kingsport, TN., has been practising a systematic approach that is designed to integrate various technical disciplines. Maintenance, operations, engineering, and analytical chemistry are all essential elements of a successful programme in process analytical chemistry and instrumentation.

The enthusiasm of the process analytical community for on-line measurements has led many to believe that the intent behind the use of process analysers is to put the analytical laboratories out of business. Is there a real case and reason for this perception, or does the analytical laboratory play a vital role in the enhanced usage of process analysers?

The relationship between the analytical laboratory and process analytical laboratory personnel should be a 'marriage'-type partnership. The successful implementation of process analytical technology relies heavily on the analytical laboratories' knowledge about process stream composition (including measurement interferences), calibration and validation issues, and troubleshooting instrument and measurement problems, etc. In addition, in this decade of resource constraints, the complementary nature/role of these two communities strengthens the sense of getting the job done in an effective and efficient manner.

During the course of this presentation, examples were cited to describe the relationship between these two communities at ECC. In addition, organizational issues that were overcome in this partnership were described.

### Analytical laboratory—Calibrate and/or verify process analysers

*Ernie H. Baughman, Dave R. Van Hare and George H. Vickers, Amoco Corporation, Amoco Research Center, PO Box 3011, Naperville, IL 60566-7011*

For many process control applications, accuracy requirements for process analysers are not high. The analyser must be precise and must properly trend with the reference method but offsets, while certaintly not desirable, are tolerable. However, when the process analyser is used to certify shipping specifications, the accuracy of the result must at least match the customer's requirements. How can one 'prove' that the process analyser result is accurate? There are two traditional approaches to this: (1) force standards into the analyser and compare the analyser results with the 'known' values; (2) take a sample to the plant laboratory and analysis by the reference method and 'correct' the analyser reading to match the laboratory value. What are the positives and negatives of these approaches? Are they different with multivariate 'model' based analysers? This presentation talk focused on the necessary role of the plant laboratory in turning final product analyser potential savings into real savings.

### Coordination of sample preparation and analysis instrumentation via supervisory software to form integrated systems

*Pete Snipes, Wayne Bullaughey, Shirley Jupiter, Chris Wurm and Steve Engel, Hewlett–Packard Company, Little Falls Site, 2850 Centerville, Road, Wilmington, DE 19808*

Bench Supervisor software provides a common user interface for sequencing samples through a variety of preparation and analysis steps. Samples may be prepared using a supercritical fluid extractor or a liquid-handling preparation station and then analysed on a GC, GC/MS, or LC. A standard instrument interface allows other instruments to be added with minimal effort.

The Bench Supervisor extends the concept of running a single instrument method on a sample to running a 'bench method' where an original raw sample may go through a preparation step that produces multiple sub-samples to be analysed. All the instruments controlled by the Bench Supervisor share a common tray of vials. A graphical user interface allows the operator to allocate sections of the tray for samples, empty vials, spe and filter cartridges, and vials containing reagents. The Bench Supervisor then manages the use of these resources as the samples are run.

This paper described the concepts of the Bench Supervisor that allow the end user to automate sample preparation and analysis on the lab bench: scheduling 'bench methods' and 'bench sequences' on multiple instruments, managing vial resources, and linking data and vials from one instrument step to the next.

### The chemist's workcell—automation strategy for sample preparation to final report

*Charles R. Knipe, Hewlett–Packard, 2850 Centerville Rd, Wilmington, DE 19808*

Since the development of microprocessors and low cost computers, chemical analysis, data reduction and report generation have all become routine automation tasks. It has only been recently with the automation of sample preparation and sample handling that full automation of all of the chemists' tasks can be carried out with no user intervention. This presentation showed how these operations can be carried out in a semi-fixed format: a 'Chemists' Workcell'. The workcell is comprised of a solid sample preparation unit (supercritical fluid extractor), a liquid handling unit, an analytical instrument and a computer workstation that co-ordinates the activities of the other components and provides report generation and networking capabilities. The workcell consists of fixed automated units that perform specific tasks and use a common preprogrammed sample translator to physically move sample and reagent vessels between the various components.

Traditional laboratories tend to segment the operations performed on a sample. In a workcell environment the chemist can select which of these tasks to perform either individually or in concert with one another. The workcell can either be controlled by an individual chemist, or with the addition of robotics and network capabilities, multiple workcells can be woven into the fabric of even larger automated systems. Several examples were discussed, demonstrating the versatility of this approach. Applications included analysis of PCBs in fish tissue, PAHs in soil, and analysis of TPHs in soil. These samples required extraction of a solid sample (using SFE), generation and analysis of calibration standards and analysis of the analytes by either GC, GC/MS or LC. Discussion included overlap versus sequential operation, batch mode versus serial mode, chain of custody control, GLP considerations and other automation concerns.

### Flexible robotic workbench for automated enzyme kinetics

*Alan B. Todtenkopf, Altotech Laboratory Systems, A Division of Sparta, Inc., PO Box 2000, Bridgewater, NJ 08807, Mark F. Russo, PhD and David J. Balaban, PhD, Sterling Winthrop Inc., 9 Great Valley Parkway, Malvern, PA 19355*

The authors described a project performed jointly by Sterling Winthrop Pharmaceutical Research Division, and Altotech Laboratory Systems to develop a workbench for automating the many tasks required when performing experiments to support the search for potential drug candidates. The workbench software allows scientists to define their own experiment sequences in a chemistry-like language and to configure their reagent layout. Furthermore, the software is easily configurable to recognize new robot peripheral devices and is thus easily setup for a wide range of different applications. A particular application of FlexBench was described.

The *hardware* component of the workbench consists of a HP ORCA robot, several measurement and auxiliary devices, and two personal computers connected by local-area-network. The *software* component of the workbench, 'FlexBench', is a family of related programs running under the Microsoft Windows environment. The set of programs consists of a 'Lab Report Editor' and several specialized editors that are invoked by 'clicking' on different portions of the lab report document. A scientist uses these editors to describe the *experiments* (protocols), *experimental setup* (equipment and reagent configuration), and a *sample batch* (rack(s) of samples and experiments to perform on each sample).

Once the information is entered with these editors, the scientist can invoke Flexbench's *Control Panel*. Here, the scientist can simulate or execute the batch. The controller also has sophisticated error recovery capabilities. FlexBench dynamically generates custom programs for the robot to execute and contains a real-time *scheduler* that allows the robot to execute assays for several samples simultaneously while guaranteeing adherence to strict timing constraints. The results produced along with all the setup information is stored in a local database thus forming an unprecedentedly comprehensive audit trail. In addition, FlexBench contains facilities for adaptive experimentation. Values in an experiment description such as concentrations or volumes can depend on the results produced in a previous experiment.

FlexBench is an extremely flexible robotic system for use by research scientists as an exploration tool as well as for repetitive screening assays. The scientists can use the robot to perform method development before finalization of an assay and robot system developers can quickly retool robots to perform new applications when old ones are no longer needed. Altotech may soon offer FlexBench as a commerical product.

### Automated soft drink analysis: A case study for non-robotic sample handling

*Dan Sullivan, Pepsi-Cola Company, Research and Technical Services, Valhalla, NY 10595*

Traditional soft drink analysis is labour intensive and too slow for modern production lines. An automated computer integrated mini-lab has been developed to eliminate manual sample preparation and data collection, and to integrate the laboratory with the production line.

The mini-lab performs all the functions of a traditional lab in a 6′ × 3′ × 6′ space. The analysis system accepts syrup, finished beverage directly from the production line, and standards for titration, density determination, and HPLC analysis. Syrup samples are diluted before analysis and finished beverage is sparged with air to remove $CO_2$. Each instrument can analyse standards to monitor instrument performance. Data is output as a report and archived for exporting.

There are a number of advantages inherent to the automated system: Since it is operator free, results are more consistent and QC staff is more effectively utilized in other plant areas. The system allows for more frequent and thorough testing, improved instrument performance monitoring, and data tracking: thus, data is more timely and complete.

### Laboratory automation: Method development and product validation for the pharmaceutical industry

*Susan G. Kelley and Arthur L. Martin, Source For Automation Inc., 115 Cedar St, Milford, MA 01757*

With the introduction of dedicated automated chemistry workstations, laboratory automation is now used routinely in pharmaceutical quality control laboratories for doing content uniformities and composite assays. Before data can actually be released, however, system validation and specific product method development and valication must be completed. Source For Automation, Inc. is experienced in automating methods for solid dosage assay for many different tablets and capsules. This paper outlined what has been learned about the general process of developing an automated method for a product and the validation of that method.

The first step in preparing to automate a content uniformity is to adapt the current manual method to the automated equipment. A general procedure has been developed which accommodates most tablets and capsules. This procedure includes deciding on dilution volumes, optimizing the extraction parameters, choosing the correct was solvent and system hardware. Then the sample prep method is written and tested. Occasionally a product will present specific challenges to method development. It may require modifications to the SDAS hardware or require a more complicated method procedure. Factors may include sample size, hardness, coating characteristics and extraction solvent composition. Several examples will be described.

Once the automated method is developed for a specific product, the next step is to validate it. The method validation protocol consists of a sequence of steps that tests the ruggedness of the method. Included are tests for injection precision, carryover and linearity, an extraction profile and a balance/dilution check. The final confirmation of the automated method is an equivalency test which compares the automated results to the manual results.

### Calibrating detector response in an EPA method using an automated system integrated to GC

*P. Castelli, M. Fogelman, W. Miles, K. Fogelman and L. G. Randall, Hewlett–Packard, Little Falls Site, 2850 Centerville Rd, Wilmington, DE 19808*

This presentation described an integrated system used for the automated generation of calibration standards and automated recalibration of the curves used to calibrate detector response as required in various EPA methods. These methods are used to determine priority pollutants in drinking water. An automated integrated system represents an alternative to current manual dilution techniques, which are tedious, error prone and time consuming.

Autosampler vial tray-based automation was utilized to make calibration standards. The calibration standards were prepared at a sample preparation station situated around the tray. After preparation, the standards were automatically injected into the gas chromatograph. Software control allowed communication and scheduling of the sample preparation process in conjunction with the control of the analytical GC.

It was demonstrated that this system was capable of both generating the calibration standards and automatic recalibration of the calibration curves. A stock solution is input to the system and five calibration solutions are created using a mixture of serial and linear dilution. For example a 0·5 mg/ml DCB stock solution is input and five standards from 0·01 ng/ml to 1·0 ng/ml in hexane were prepared. Typical values for the calibration curve linearity were discussed and some results representative of the EPA method assays were presented.

### Trends in automated sample preparation: SFE and SPME

*Ilona Davies, Elizabeth Almasi and Zelda Penton, Varian Chromatography Systems, 2700 Mitchell Drive, Walnut Creek, CA 94498*

The determination of trace-level volatile and semi-volatile pollutants in environmental samples is usually a difficult task. Automated sample preparation greatly simplifies this task by concentrating or isolating the target components from the complex sample matrix prior to chromatographic analysis. Supercritical fluid extraction (SFE) and solid phase micro extraction (SPME) are unique and complementary sample preparation techniques for a wide range of environmental samples: gases, liquids, and solids.

Automated SPME involves the extraction of volatile and semi-volatile analytes from a matrix using a fused-silica fibre which is coated with stationary phase and mounted in a GC autosampler. The fibre may be immersed directly in a liquid material, such as waste water, or positioned within the headspace region of a solid material, such as a polymer. SPME is able to duplicate sample preparation currently done by purge-and-trap, headspace, solid phase extraction (SPE), and liquid/liquid extraction techniques.

On the other hand, SFE uses a supercritical fluid to vigorously extract trace-levels of volatile, semi-volatile and even non-volatile compounds from solid samples, such as soils, foods, and air particulates. Automated SFE using non-toxic supercritical carbon dioxide is safer, faster, and far more efficient than traditional Soxhlet extraction methods.

Automated SPME and SFE complement each other and fulfill many of the sample preparation requirements for the average environmental laboratory. The wide range of potential applications of SPME and SFE with GC or GC/MS were discussed.

### Enhanced sample handling automation for sample concentration

*Mark Cava and David S. Williams, Zymark Corporation, Zymark Center, Hopkinton, MA 01748*

Oversupply and a weak business cycle for environmental lab services has forced consolidation and a need for performance enhancement. Today, the biggest gains in efficiency take place in the organic extraction laboratory. Performance enhancement for sample extraction and concentration requires change. To change, laboratories let their best people lead implementation of sample automation. The result is a dramatic improvement in operating efficiency.

TurboVap Concentration Technology has revolutionized laboratories by providing consistent results, low cost per sample, and twice the throughhput. Payback periods of less than three months are normal for TurboVap.

The AutoTrace Workstation has turned solid phase extraction into the preferred extraction method for huge savings in solvent and labour. Payback periods for AutoTrace ranges from six months to twelve months.

Automation of technique-dependent sample concentration steps provides improved reproducibility, accuracy, and the fast turnaround needed for integrated laboratory management. TurboVap and AutoTrace Workstation automation for sample concentration of drinking water, groundwater, waste water, and soil will be discussed. Results are for compounds regulated under Resource Conservation and Recovery Act (RCRA), National Pollution Discharge Elimination System (NPDES), and Safe Drinking Water Act (SDWA). Target groups include semivolatiles, pesticides, explosives, herbicides, PAHs and PCBs. The automation methods, GC results, HPLC results and payback were examined.

### Automated preparation and derivatization of selected analytes employing integrated sample preparation and GC/MS analysis

*Wayne S. Miles, L. G. Randall, Patricia Castelli, Kimber Folgelman and Marla Folgelman, Hewlett–Packard, Little Falls Site, 2850 Centerville Rd, Wilmington, DE 19808*

Chemical derivatizations have been widely used to enhance analyte activity in a variety of chromatographic applications. Manual sample preparation procedures involving derivatization steps are tedious and time consuming, and have potential sources of error resulting from user intervention. The ability to automate sample preparation is attractive to the user since it provides among other things, less hands-on time per sample, precision in manipulation, fewer opportunities for error, as well as reduced exposure to hazardous samples and chemicals. Another advantage of automation is the 'just-in-time' delivery of samples for analysis. The result is no loss in activity of reacted sample due to breakdown of product. With a fully automated system like the HP7686 Autosample PrepStation Sample Preparation Module, complete sample preparation including derivatization can be achieved with the benefit of improved accuracy and precision.

In this paper methods for automating manual sample preparation and derivatization procedures were discussed and results for analyte derivatization by acylation, alkylation, and silylation were presented. This approach was applied to derivatization of carboxylic acids, amines, and selected NIDA drugs of abuse. The automated derivatization were performed as the last step in the extraction of analytes by automated solid phase extraction. It is important to note that the entire analytical method (sample prep and analysis), was carried out on an integrated sample preparation analyte detection GC/MS system. Recovery and completeness of reaction data for these applications show that automated derivatization coupled to preparation and analysis greatly improves the analytical integrity of the results. For example, CVs for the quantitation of Benzoylecognine were in the range of 1–3% at levels of 40 to 2000 ng/ml.

### Instrumentation for automated supercritical fluid extraction

*Raymond K. Houck, Doug Koebler, Glen Williams and Joseph M. Levy, Suprex Corporation, 125 William Pitt Way, Pittsburgh, PA 15238*

The use of supercritical fluid extraction (SFE) has evolved as an alternative sample preparation tool for many applications. Distinct advantages compared to liquid solvent extraction of speed, efficiencies, reliability and selectivity can further be enhanced by automating the SFE process.

In this presentation, a new sequentially automated SFE system and process was presented. The automation of the SFE process includes the sequential freeing of extraction vessels, the ability to process samples overnight and the flexibility to change pressure, temperature, extraction fluid composition, extraction fluid flowrate, vessel size and collection strategies for each sample. Thus the system has both the flexibility for automated methods development and the throughput for high volume, unattended overnight operation. Moreover, a micro-processor controlled decompression device which automatically controls extraction flow rates ensures consistent and reproducible operation. Details exemplifying these instrumental features were presented with specific examples in environmental, food and polymer applications.

### Using flow injection to improve graphite furnace accuracy and detection limits

*Glen Carnrick, Susan McIntosh and Christoper Hanna, Perkin Elmer Corp., Norwalk, CT 06859-0219*

While modern graphite furnaces permit the direct analysis of a wide variety of complex samples, there is a practical limit to the amount of matrix that can be tolerated. Matrix volatilized along with the analyte can produce high levels of non-specific absorption, resulting in a degradation of the signal-to-noise ratio. Additionally, covolatilization of matrix and analyte can result in incomplete recovery of analyte due to gas phase recombination of the analyte and matrix, or from reduced analyte residence time due to the gas expansion of the matrix. Some of these matrix related problems can be reduced or eliminated by utilizing furnace pyrolysis and matrix modification to remove some or all of the matrix prior to analyte atomization. An alternative approach is to use ion-exchange or solvent extraction to remove the matrix from the sample prior to injection into the furnace. The disadvantage of these techniques is that they are time consuming and a potential source of contamination. For elements such as As and Se, hydride generation may be a preferable technique to separate the trace metals from the concomitant matrix.

In this presentation, the authors described a fully automated system for the determination of hydride forming elements that uses a standard flow injection system directly coupled to a transversely heated graphite furnace. The volatile hydrides are introduced through the dosing hole of graphite tube, decomposed at temperatures of 200–400°C and trapped and concentrated on a platform that has been treated with Ir. Since much larger sample volumes may be used with flow injection than in conventional graphite furnace, the resulting detection limits may be up to two orders of magnitude lower than those obtained with other conventional graphite furnace techniques.

Interferences from halides and sulphates in the graphite furnace were compared with those obtained using the flow injection-hydride generation furnace technique. Results obtained for the analysis of a variety of heavily matrixed samples such as sea and estuarine waters were discussed. Detection limits were compared with conventional furnace techniques.

### Design and implementation of expert systems to aid HPLC troubleshooting

*John W. Dolan, Lloyd R. Snyder and Robert W. Albrecht, LC Resources Inc., 2930 Camino Diablo #110, Walnut Creek, CA 94596*

Efficient identification and correction of problems related to liquid chromatographic (LC) separations has traditionally been an area that has required many years of experience. Whereas simple hardware problems such as leaks or overpressure can be handled with comparatively little knowledge of chromatography, chromatographic problems such as peak tailing, may frustrate even the experienced chromatographer.

Traditional troubleshooting aids include personal experience, books and troubleshooting guides, as well as the use of in-house experts. The goal of computerized expert systems for LC troubleshooting is the encapsulation of available expertise in a form that will enable the relatively inexperienced chromatographer to solve problems that have historically been left to the experienced professional. To be of practical use, the user interface for such a system must be designed so that it is natural and efficient to use. User interactions with the system must be straightforward and not redundant.

In terms of implementation, the simplest computerized systems directly query the user for all of the required data. At the other extreme are expert systems that interact with the LC hardware, discovering and correcting errors with little or no user interaction.

### Computer-assisted evaluation of differences in LC column selectivity

*Janice Lewis[1], John Palmer[2] and Thomas Jupille[1], [1] LC Resources Inc., 2930 Camino Diablo #110, Walnut Creek, CA 94596, [2] Mac Mod Analytical, 127 Commons Ct, Chadds Ford, PA 19317*

Liquid chromatographers have long recognized that columns of different surface chemistry could provide useful differences in selectivity for particular analyses. Until recently, this phenomenon has been underutilized because of the tremendous amount of experimental work required to characterize column selectivity and its changes with mobile phase composition. As a result, many LC separations are carried out on the familiar alkyl-bonded phase columns when they might be significantly improved by a change in column type.

A computer-modelling approach allows the selectivity of any column with regard to solvent composition to be mapped on the basis of only two or three experimental runs. This allows a range of column types (for example C8, phenyl, and cyano) to be screened for suitable selectivity in a reasonable working period of a day or so.

This technique can be extended to evaluating column-to-column variations or to compensate for changes in selectivity resulting from column degradation or aging.

### A new data system interface design for automated HPLC

*James A. Schibler, J. O. Adams and Der-Min Fan, Dionex Corporation, 1228 Titan Way, Sunnyvale, CA 94088-3603*

Analytical instruments, such as liquid chromatographs, are available either as integrated systems or as modular components. Each option has its advantages, but the modular approach is gaining popularity because it can more readily adapt to the increasing rate of change in today's laboratory.

A critical issue for the user of a modular system is how well the modules work together, since modules are not always designed at the same time. Typically, the modules are integrated with a central control interface connected to a personal computer (PC). Unfortunately, this scheme has several drawbacks:

(1) All communications are limited by the speed and functionality of the interface between the instruments and the PC.
(2) Modules must be typically located near the central interface, limiting flexibility.
(3) Only limited status information is typically available from each module, which makes documentation of run conditions for regulatory compliance difficult.
(4) Costly, time-consuming redesigns of interface hardware and/or firmware are required to support new functions or modules.

This presentation discussed the computer interface design of a new modular line of HPLC instruments. In the new design, the HPLC modules and host computer communicate directly with each other using IEEE 802.3 (Ethernet) protocol. Advantages of this design over conventional approaches were:

(*a*) Increased speeed and bandwidth of communications.
(*b*) Greater flexibility in module locations and configurations.
(*c*) Easier modification for future enhancement.
(*d*) Complete documentation for compliance with Good Automated Laboratory Practices.

### The determination of acetaminophen by flow injection Fourier transform infra-red spectrometry

*Maiella L. Ramos, Julian F. Tyson and David J. Curran, Chemistry Department, University of Massachusetts, Amherst, MA 01003*

A quantitative study has been undertaken of the possibilities for the determination of species with IR active vibrations in aqueous solutions by flow injection (FI) Fourier transform infra-red (FT-IR) spectrometry. A single line FI manifold was used to present aqueous sample solutions to the spectrometer equipped with a micro version of the cylindrical internal reflectance cell for liquid evaluation (the so-called CIRCLE cell).

Acetaminophen samples were dissolved in a buffer solution of 0·05 M $NaHCO_3$ and 0·1 M NaOH at a pH of 9·8. A continuous flow mode was used for quantitative analysis of the samples by using the CIRCLE accessory. An optimization study was carried out in which the effects of several parameters have been studied. These included the flow rate, sample injection volume, number of sample scans, mirror velocity, and resolution. The signal to noise ratio depends on these parameters and the optimum values of the variables were found to be 0·5 ml/min, 158 μl, 12 sample scans, mirror velocity of 50 KHz and resolution of 16 $cm^{-1}$. Quantification was carried out at the phenolic (OH stretch) band at 1275 $cm^{-1}$ in aqueous solutions, for which the working range studied was 0·0005 M to 0·013 M. The FT-IR provides a continuous monitoring of the spectral baseline which permits an accurate determination of the peak maximum in the absorbance band.

The ability of the FT-IR system to collect data for monitoring time-dependent processes by the use of a software package called Time-Evolved Kinetics Operations (for fast kinetics) was evaluated. The possibilities for extending the procedure to the monitoring of on-line chemical derivatization were also discussed. The analytical potential of the FIA/FT-IR combination was critically evaluated.

### Standardizing the automated environmental chemical laboratory

*Robert M. Hollen, Los Alamos National Laboratory, PO Box 1663 MS J580, Los Alamos, NM 87545*

Automation of the technology and practice of environmental laboratory analysis has not been as complete as one might expect. Confined to autosamplers and limited robotic systems, our ability to apply production concepts to environmental analytical analysis is not extensive. With the impending remediation of the governments hazardous waste sites in the United States, only the application of production chemistry techniques will even begin to provide those responsible with the necessary knowledge to accomplish the cleanup expeditiously and safely. Tightening regulatory requirements have already mandated staggering increases in sampling and characterization needs with the future only guaranteeing greater demands. The Contaminant Analysis Automation (CAA) Program, managed by the Department of Energy's (DOE) Office of Technology Development, has as its mission addressing these current and future characterization needs by the application of a new robotic paradigm for analytical chemistry.

By designing and transferring to industry systems based upon the Standard Analysis Method (SAM) architecture, the CAA group is working towards a solution. Each SAM system will automate a specific environmental chemical method, from sample preparation through analytical analysis. It then generates knowledge of the remediation site via sophisticated knowledge-based data interpretation. The building blocks of a SAM are standardized modules, hardware and software, that automate a sub-protocol of an analysis method. This concept allows the chemist to assemble an automated environmental system without the worry of incompatibility. Hardened for the rigors of on-site remediation, these systems will be designed with use within transportable laboratories directly at the remediation site.

### A control system for modular automation of chemistry

*J. Michael Griesmeyer and Timothy D. Urenda, Sandia National Laboratories, Intelligent Systems and Robotics Center, Department 2161, Albuquerque, NM 87185-0952*

The basic approach to automated laboratory system control in the DOE Contaminant Analysis Automation (CAA) Project was described. Standard Laboratory Modules (SLMs) are 'plug and play' building blocks for the automated laboratory. Each SLM can perform a subset of the operations required to implement complete analysis methods. A method is programmed as a hierarchical script of operations that can be performed by the individual SLMs. The Task Sequence Controller (TSC) processes these scripts by expanding them into primitive operations that can be performed by the individual SLMs. The TSC being implemented at Sandia is modelled after supervisory workcell control systems used in manufacturing environments. Process scripts are associated with samples before they are introduced into the laboratory. Analysis information is, therefore, independent of the individual laboratory that processes it. This notion of a 'smart sample' allows for complete flexibility in laboratory system integration. SLMs may be added, removed, or replaced at any time without disruption of lab operations.

Parallel execution of the process scripts accelerates the flow of samples through the laboratory. Scripts provide for branching of operations that may depend on conditional determinations made during processing. This allows for complex logic to be embodied in the method. For instance, a GC screening operation may determine the concentration of a sample and indicate the need for further concentration or dilution.

Additional benefits of the CAA laboratory include the repeatability of analyses and the automation of data handling. Parameterized method scripts allow for variations needed to satisfy customer requirements. Once a method script is developed, any sample associated with that script will be analysed in the same way. Results from each of the sample preparation and analysis steps are recorded to provide a completely automated audit trail. Data can be recalled and formatted to generate any type of report.

The key to modular chemistry is standardized interfaces between components of the automated laboratory. The CAA Project has developed an interface specification that defines protocols for interaction between any SLM and the TSC, as well as interaction protocols for other software components. The authors are implementing a testbed of hardware and software modules to test the interface protocol specifications.

### Data interpretation in the automated environmental analysis laboratory

*Leon N. Klatt, Robotics & Process Systems Division, Oak Ridge National Laboratory, PO Box 2008, Oak Ridge, TN 37831-6303, John W. Elling and Wesley P. Unruh, Sensor Systems and Robotics Group, Los Alamos National Laboratory, P.O. Box 1663, MS J580, Los Alamos, NM 87545*

A fully automated environmental analysis laboratory will require automated sample preparation, automated instrumental analysis, automated interpretation of the data obtained from the analytical instrument, and automated quality assurance review of the analytical result. As part of the US Department of Energy's Office of Technology Development, Contaminant Analysis Automation (CAA) Project, Oak Ridge National

Laboratory and Los Alamos National Laboratory are working to define the generic technical issues and needs for the automated interpretation of data. The concept being developed involves an expert system driven Data Interpretation Module (DIM). In the context of the CAA automation concepts, the DIM will be a Standard Laboratory Module (SLM) that interfaces with the automation Task Sequence Controller (TSC) through the standard SLM software interface. The proposed DIM will operate in two modes: an on-line mode and an off-line mode. In the on-line mode the DIM will operate autonomously and under the control of the TSC. The off-line mode will allow the analytical chemist to build automated data analysis methods and the requisite calibration functions. The expert knowledge of the analytical chemist will be encapsulated into the knowledge base of the expert system. Pattern matching data processing tools will be an important part of the DIM. This paper described the approach being taken in the development of the DIM, and presented some results based upon an initial implementation of EPA Method 8080 'Organochlorine Pesticides and PCBs' as a target protocol.

### Electronic laboratory notebooks

*Raymond E. Dessy, Chemistry Department, Virginia Polytechnic Institute and State University, Blacksburg, VA 24061-0212*

The technical issues associated with the creation of suitable Laboratory Electronic Notebooks have largely been solved. Stylus driven input, identification, encryption and storage, and network sharing are realities. The benefits to the scientist are obvious. Automatic indexing, full text search, compound document architecture, automatic report generation, and network sharing have been done in our labs and others. The legal issues with respect to court acceptability and litigation search boundaries have been addressed. What is going to be more difficult are the recognition of, and solutions to, issues involving people and the changes in work habits required to make the tool effective.

How does one make the notebook understandable to others? What materials are shared and when? What new tools will scientists need to handle the large amount of material now available to them? How are live transfers to reports handled? How are reinterpretations redistributed throughout the network? Little effort has been expended on the psychological factors involved in a new medium of recording, storage, and distribution. It is not enough to merely emulate what has been the practice on cellulose; indeed that is the worst type of implementation. The Laboratory Electronic Notebook should recognize that new tools require an efficient match between the human being and the inanimate, but interactive, tool itself.

This presentation focused on many of the human factor issues involved, as well as presenting a background to the area and providing a vocabulary for the technology that will support Electronic Laboratory Notebooks.

### *In vivo* sensing using ion-selective electrodes

*Richard P. Buck, Vasile V. Cosofret and Erno Lindner, Department of Chemistry, University of North Carolina, Chapel Hill, NC 27599-3290*

Development of flexible, polyimide-based array microsensors for redundant sensing of a single ion activity, spatial resolution of ion activities, or for sensing of several ion activities in defined spatial relations, has progressed in *in vivo* testing. This work now includes investigating systematic calibration procedures, *in vivo* biocompatibility measurements using impedance and electron micrography with sampling of adhering materials, and chronic applications of the electrodes inserted into pig and dog hearts.

The new results are based on *in vitro* calibrations, stability of slope and intercepts and *in vivo* comparison or our electrodes with glass electrodes in the same animal. Applications are acute and so do not require detailed calibrations after implantation. Subsequent *in vivo* testing uses massive injection of acid or salt solutions. Efficacy of the electrode function for chronic measurements is deduced indirectly over 14 days implantation using impedance measurements, leukocyte counts, and micrographs of the biological rejection process showing phases of cell activity in comparison with the changing resistive component of the impedance. Unpublished work stresses the microelectrodes coated with Ag/AgCl and continues with studies of complete electrodes.

New results lead to tentative conclusions on the optimum compositions of polymer and plasticizer for *in vivo* ion sensor construction up to the final protective coatings that require ion-transport as well as inertness and mimicking of exterior cell bilayers.

### Current status and future prospects for commercialization of implantable biosensors

*Kirk W. Johnson, Douglas J. Allen and Charles C. Andrew, Lilly Research Laboratories, Eli Lilly and Company, Indianapolis, IN 46285-0815*

Many novel biosensors have appeared in the literature over the past 35 years. Some radically different, others just a slight change from its predecessors. In this manner, the implantable biosensor has evolved to where it stands now. Surprisingly, the market is not flooded with these wonderful little devices which were projected to revolutionize medical care.

This paper summarized the research and development process required for an implantable biosensor. Specifically, the glucose sensor designed for subcutaneous implantation developed by Lilly was used as an example. Technical and non-technical issues were discussed.

Once a novel form of implantable biosensor is discovered, the commercialization of the sensor is contemplated. Technical, medial, financial, and marketing issues are all considered at this point. Unfortunately in this day and age, an obvious medical need is not enough of a reason to commercialize a product.

Technical issues are very important, particularly mass-production of the sensor. The ability to produce a functional, reliable implantable biosensor at the rate of thousands per day is a far cry from the researcher making several per day with a success ratio of 50% at best. This process requires very expensive equipment and elegant processes. Other technical issues such as biocompatibility of the sensor during long-term implantation, lifetime and stability of proper functioning, and method of implantation must also be overcome.

Issues pertaining to FDA regulatory requirements must not be overlooked. This very important step in the development process consumes a great amount of time and money. Marketing issues such as a sales force, packaging, and what segment of the maket to target are also considered. The actual cost of the research, development, mannufacturing and marketing of the sensor is calculated and a selling price is projected.

In retrospect, the technical problems that were overcome during the research phase of this project in order for the implantable glucose sensor to function properly are just the 'tip of the iceberg'. Commercialization of an implantable biosensor, like many other medical related products such as pharmaceuticals and medical equipment, is a very difficult and expensive process.

In conclusion, the transition from an implantable sensor discovered in a laboratory to an implantable sensor actually on the market is a quantum leap. Great care must be taken in the development of these devices due to the liabilities associated with the medical information they supply.

### Nafion dryer tubes for moisture removal in flow injection cold vapor/hydride generation atomic absorption spectrometry

*N. G. Sundin, J. F. Tyson, Department of Chemistry, University of Massachusetts, Amherst, MA 01003, S. McIntosh, and C. P. Hanna, Perkin-Elmer Corporation, 761 Main Avenue, Norwalk, CT 06859*

Mercury and the metalloid elements are most often determined by the cold vapour/hydride generation technique. This method involves the use of reductants (tin(II) chloride or borohydride), to reduce the analyte to a gaseous product. Elemental mercury or a hydride is then stripped from solution in a gas–liquid separator using an inert carrier gas and directed to an atomic absorption spectrophotometer. Many workers have noted problems such as loss of sensitivity and blockage of transfer lines due to excessive moisture transported to the atom cell or lodged in the transfer line. This work will demonstrate one way to continuously remove the water vapour through the use of semi-permeable Nafion dryers.

Water is removed by passing a dry sheath gas around the outside of the hydroscopic Nafion membrane transfer line. These Nafion dryers remove water at the rate of 1·7 mg/min at greater than 90% efficiency when $SnCl_2$ is used as the reductant for Hg determinations. No measurable change in precision is observed and only a slight loss in sensitivity (due to the larger internal volume of the dryer versus standard Teflon tubing) is seen. The Nafion dryer can remove up to 4·9 mg/min of water vapour at a temperature of 60°C. Even at this temperature the efficiency of water removal remains over 90%. An amalgam trapping device was used to show that the loss of Hg vapour through the Nafion membrane was less than 0·04%.

This Nafion membrane can also be used to remove water vapour when borohydride is used as the reductant. In this case, 2·3 mg/min of water is removed at an efficiency of 91% at a borohydride concentration of 0·4%. Only a slight loss in sensitivity is observed for the determination of arsenic.

### Using flow injection techniques to expand the capabilities of a flame atomic absorption spectrometer

*Charles A. Schneider and John T. McCaffrey, Perkin-Elmer Corp., 761 Main Ave., Norwalk, CT 06859*

Flow injection flame atomic absorption can significantly expand the applications of flame AA. In addition to improving the analytical results, flow injection can be used to automate many routine tasks to increase lab productivity and better utilize analyst time.

Flow injection techniques can be used to automatically dilute samples to extend the working range and increase sample throughput. In addition, flow injection can be used to overcome problems that occur during the analysis of samples containing large quantities of dissolved solids. High dissolved salt concentrations can cause clogging of the burner slot, which can degrade analytical precision and accuracy. The use of flow injection offers real advantages for these types of samples. By injecting a small volume of sample into a continuously following carrier stream, the nebulizer is continuously rinsed. This makes it possible to directly aspirate samples that have very high levels of dissolved solids. A lower limit of detection can be achieved since no dilutions are necessary.

To highlight the benefits of combining flow injection and flame atomic absorption, the analysis of lead in paint was discussed. Various digestion procedures are currently used for this analysis. The final digestion product may have very high lead concentrations and a high level of dissolved solids. Traditionally, these sample types have created burner slot clogging problems. By using flow injection, the dilution step of the analysis can be automated and the clogging problems eliminated. Manifold design, set up and operation were discussed. The effect of flow injection on the precision and accuracy of the analytical results were explored.

Results were shown for lead in paint chips. The results were compared with traditional analytical methods. The effect of flow injection on the precision and accuracy of the analytical results was explored. Quality control procedures published by the United States Environmental Protection Agency for the National Lead Laboratory Accreditation Program were followed.

### Evaluation of chromium speciation by flow injection flame atomic absorption spectrometry

*P. Yehl, J. F. Tyson, Department of Chemistry, University of Massachusetts, Amherst, MA 01003; C. P. Hanna and S. McIntosh Perkin–Elmer Corporation, 761 Main Avenue, Norwalk, CT 06859*

The procedure developed by Sperling *et al.* has been critically evaluated and some practical problems addressed. The method is based on the selective retention of either Cr(III) or Cr(VI) on alumina. The species retained is governed by the pH of the carrier stream; Cr(III) is retained at pH 7·0 (phosphate buffer), while Cr(VI) is retained at pH 2·0 (potassium chloride/hydrochloric acid). After loading, the desired Cr species, was then eluted by passing either 1·0 M nitric acid for Cr(III) or 0·5 M ammonia for Cr(VI). The procedure was reported to increase the sensitivity for Cr detection by a factor of 25. The wash solutions suggested, 0·5 M ammonia for the removal of unwanted residual Cr(VI) during the determination of Cr(III), and deionized water for the removal of unwanted residual Cr(III) during the determination of Cr(VI), gave rise to distorted peak shapes. It is thought that this is due to a change in the surface charge on the alumina. If the buffer solutions are used also as wash solutions, an increase in sensitivity (approximately 2–3 fold) is obtained for Cr(III), whereas roughtly equivalent sensitivity is obtained for Cr(VI). The modified method increased the sensitivity from 25% of the published value for Cr(III) to roughly 50–60% of that published. Cr(VI) sensitivity was essentially unchanged, staying at approximately 60% of the published value. However, when determining 200 ppb Cr(VI) in the presence of 500 ppb Cr(III), it was found that using the loading buffer as a wash solution was not an efficient means of removing residual Cr(III) prior to elution of Cr(VI). Deionized water, proposed originally, performed this task more efficiently. Problems retaining Cr(VI), because of the limited buffer capacity of the Cr(VI) loading solution (HCl/KCl) in acidic solutions, were encountered. The Cr(VI) method was susceptible to sulphate interference. At 400 ppm sulphate, the signal depression of 100 ppb Cr(VI) was 90%. At 20 ppm sulphate, signal depression was still about 10%. Phosphate also interfered with Cr(VI) to an extent greater than reported (100 ppm and 40 ppm gave signal depressions of 83% and 21%). Recoveries of Cr(VI) of 86% from drinking water and 64% from pond water at the 100 ppb level were obtained. The pH of the carrier is crucial. Differences of as little as 0·1 pH units can affect the uptake by as much as 30%. Careful monitoring of this pH is important. There were no problems in determining Cr(III) in moderately acidic or basic solution. However, Fe(III) interfered with the Cr(III) signal to a significantly greater extent than that reported. Even at concentrations as low as 1 ppm Fe(III), 10% signal depression of a 100 ppb Cr(III) sample was found. Recoveries of Cr(III) were 71% from drinking water and 72% from pond water at the 100 ppb level. It was possible to determine 200 ppb Cr(III) in the presence of 500 ppb Cr(VI) efficiently with the revised wash solution. The linear calibration range was from 10 ppb to 200 ppb for both species. Using different cartridges on equivalent samples under identical conditions, signals varied by as much as 30%. The lifetime of the cartridges was variable, and appeared to be related to the nature of samples analysed with them. The method developed has a significantly greater capacity and efficiency for Cr(III) than for Cr(VI).

### Advancements in automation of quality control procedures for all laboratory requirements for atomic absorption spectrometry

*Christine Flajnik, Fred Delles, Varian OSI, 201 Hansen Court, Wood Dale, IL 60191 and Brian Frary, Varian Australia, 670 Springvale Road, Mulgrave, Victoria 3170, Australia*

This paper described new generation, automated, real time quality control software to be utilized in any laboratory setting for the validation of data. Thirteen tests are available for selection. Each test has its own selectable limits. They can be activated by sample frequency or random placement by label coding to allow for a customized system. Time limit overrides are provided to keep the data valid. Specific error actions can be selected for each required test. Practical applications of the system for flame and furnance analyses were discussed.

### Optimization of the design concepts for vapour generation coupled to atomic fluorescence measurement for ultratrace determination of the hydride forming elements and cadmium

*Peter B. Stockwell, Warren T. Corns, P S Analytical Ltd, Arthur House, B4 Chaucer Business Park, Watery Lane, Kemsing, Sevenoaks, Kent TN15 6QY, P. Goodall, S. Hill and L. Ebdon, University of Plymouth, Drake Circus, Plymouth, Deven PL4 8AA*

The advantages of atomic fluorescence (AFS) over other techniques are well established and are ingrained on students of analytical chemistry in their training. It is very surprising that AFS instruments are not generally available. Recently these techniques have found greater appeal when coupled to pretreatment technologies such as vapour generation. This paper outlined the advantages of this coupled technique and described the necessary optimization to maximize the application for each element. Broadly speaking, the classification can be made according to vapour generation, hydride generation and organic complex vapour generation.

Mercury is determined to the first criteria arsenic, selenium and antimony by the second and cadmium/lead by the third.

Table 1 outlines the differences and similartieis of each of the three types of detectors.

The application of three distinct instrumental configurations to analyse these elements were outlined. Results were presented showing excellent reproducibility and low detection levels.

A range of certified reference materials has been successfully analysed which shows the flexibility and

*Table 1. Comparison of Vapour Generation—Atomic Fluorescence Spectrometry Techniques (Stockwell et al.).*

| Analyte | Generated species | Excitation source | Filter | Atom cell | Detector |
|---|---|---|---|---|---|
| Hg | Mercury vapour | Low pressure vapour discharge lamp | 254 ± 10 nm | Flow cell | UV-VIS PMT |
| As | Covalent hydrides | Boosted discharge hollow cathode lamp | Multi-reflectance filter 190–220 nm | Argon— Air Hydrogen Flame from reagents | Solar-blind PMT |
| Se | | | | | |
| Sb | | | | | |
| Te | | | | | |
| Cd | D1-ethyl compound | Vapour discharge lamp | 228 ± 10 nm | Argon— Air Hydrogen flame from cylinder | Solar-blind PMT |

versatility of the detectors. Each of these are capable of determining the elements of interest in the parts per trillion region.

### Flow injection atomic fluorescence spectrometry

*J. F. Tyson and P. Yehl, Department of Chemistry, University of Massachesetts, Amherst, MA 01003*

Atomic fluorescence spectrometry (AFS) is a sensitive and selective spectroscopic technique which, because a selective nature of the excitation is possible, minimizes spectral interferences from other elements. The availablity of commercial instrumentation is somewhat limited due to the competitive performance of atomic emission spectrometry. However, it is considered that AFS based on hollow cathode lamp light sources and an inductively coupled plasma atom source (as performed with the Baird Atomic AFS 200 and 2000 instruments) has considerable potential as a multi-element detection mode for flow injection and chromatography. This potential has been evaluated for the determination of elements for which chemical vapour generation is normally used (such as arsenic, selenium and mercury). The basic characteristics of the instrument for flow injection detection have been evaluated and the effects of the usual FI operating parameters, flow rate, volume injected, manifold configuration have been shown to exert the same influence as for flow injection introduction to plasma optical emission instruments. Detection limit characteristics and freedom from spectral inferences (as typified by aluminium on calcium and zinc/copper overlap) were determined, as were the extent of EIE effects. The possibilities for removal of these by selection of suitable operating conditions were assessed. The potential of the technique of FI-ICP-AFS for use in conjunction with solid phase extracts for the determination of CrIII and CrVI and for the determination of gold were assessed. It is considered that the procedures could be applied to the analyses of some environmental, biological and gelogical samples. The possibilities for use as an element specific detector for chromatography was evaluated for speciation studies involving lead, tin and manganese.

### Environmental applications of pattern recognition Techniques

*Barry K. Lavine, Box 5810, Department of Chemistry, Clarkson University, Potsdam, NY 13699-5810, Howard Mayfield and Abdullah Faruque, AL/EQC-QL, 139 Barnes Drive, Suite 2, Tyndall AFB, FL 32403*

The technique of gas chromatography/mass spectro metry (GC/MS) has been used by the United States Air Fource to identify environmental pollutans, for example jet fuels leaking from underground tanks or pipelines. Typically, identifications are made on the basis of fingerprint patterns in GC/MS data. The characteristic nature of GC/MS data suggests application of pattern recognition techniques to render data interpretation into a more quantitative form and to allow for more accurate predictions of unknowns.

AL/EQC-OL has initiated an in-house research programmme to examine samples of various jet fuels in order to develop a suitable data base of gas chromatograms representative of the different types of jet fuels. Using conventional pattern recognition techniques, AL/EQC-OL scientists have shown that fuels can be identified as to type on the basis of GC/MS data. However, jet fuel spill samples can be a combination of two or more different types of fuels. The action of the environment is another complicating factor. Conventional pattern recognition techniques cannot properly treat this type of data. Hence, the development and implementation of new pattern recognition techniques that can cope with this type of data has been a major thrust of the project. The fuzzy c-varieties clustering algorithm, FCV-false colour data imaging, and artificial neural networks are our response to AL/EQC-OL unique data analysis problems.

In this presentation the authors described the development of pattern recognition system for the identification of weathered or unweathered fuel samples from GC/MS

profile data. The proposed pattern recognition system for fuel spill identification possesses four key attributes. First, the system has the capability to assign an unknown to a fuel category with some accompanying statement about reliability of classification. Second, the pattern recognition system has the capability to flag outliers. Third, the pattern recognition system can allow the user to add new samples to the training set. Fourth, the system can identify binary or ternary fuel mixtures. The proposed system is flexible and robust enough for researchers but sufficiently user friendly for use by non-expert technicians. An anticipated application of the system is be the identification of the type of jet fuel present in a recovered environmental sample.

### Automated analysis of complex infra-red spectra using pattern recognition

*Dawei Qian, Department of Computer Science, Central Michigan University, Mt. Pleasant, MI 48859; Martin L. Spartz, Department of Chemistry, Central Michigan University, Mt. Pleasant, MI 48859; Jess S. Eldridge, Gary D. Hipple, William K. Reagen and Jeffrey W. Stock, 3M Environmental Laboratory, St. Paul, MN 55133-3331*

An automated infra-red spectral analysis system has been developed and is being tested for the near-real-time analysis of complex infra-red spectra. This system consists of three parts: (1) training set construction, (2) dimension reduction and noise control, and (3) sample analysis. This system can be used for the near-real-time analysis of infra-red spectra.

The training set is formed by supervised learning based upon standard gas phase spectra. To provide a very robust analysis program, several special techniques are applied. About 110 compounds are included in a library, a peak picking algorithm is used to select the compounds needed in the training and analysis set. This method is used to reduce the dimensionality of the error from the training set spectra. When new compounds are added, the system automatically can add the compound to the library for future use. To avoid the overestimation caused by the $H_2O$ and $CO_2$, selected frequencies are selected for the training set.

The second procedure in this system is to reduce the dimension of this training set by principal components analysis. Since the magnitude of absorbance for each compound can be quite different, the analysis is implemented through several layers in conjunction with filtering. Data scaling and weighting techniques are also used in this system.

When the above parts are constructed, the system is used for the near-real-time analysis of infra-red spectra. The sample spectra are first preprocessed for drift correction and noise reduction using the routines provided by this system. Then the sample spectra are analysed to determine the concentrations of the compounds present.

The analysis software is written in C language on an IBM compatible 486 microcomputer. Initial test results have demonstrated that if the training set used for the principle components analysis is too large errors in the reference spectra can dominate the analysis. Also, if water and carbon dioxide bands are not addressed they will describe a large part of the spectral variance. Thus, a limited training set is selected for each data collection. This software was discussed along with the many features implemented to allow for automated analysis of open-path infra-red spectra.

### Determination of main reaction products formed during hydrolysis of sugars in water by evolving factor analysis

*F. O. Libnau, A. A. Christy and O. M. Kvalheim, Department of Chemistry, University of Bergen, N-5007 Bergen, Norway*

The power of sequential rank analysis in resolving complex infra-red spectra of components in mixtures was demonstrated. The inter-conversion of the pure anomeric forms of $\alpha$- and $\beta$-D-glucose in aqueous solution was followed by means of FT-IR spectroscopy as a function of time.

The mutarotation was investigated at 22°C. The sugar solution was prepared to contain approximately 0·35 mol/l D-glucose in water. The spectral variation in the ATR-spectra in the region $\sim$950–830 cm$^{-1}$ was used to resolve the complex spectra of the two anomers of D-glucose in the region $\sim$1200–950 cm$^{-1}$. The lack of clear selective regions in the spectra is an obstacle in obtaining the concentration profiles for the two anomers. Sequential rank analysis of the first-order differentiated spectra (differentiated in wavelength direction) was used to locate candidates for the spectral optima for the pure anomers and the appropriate concentration profiles were then obtained therefrom.

In order to obtain relative concentrations, the resolved spectra were scaled relative to the isosbestic points and the concentration vectors calculated by least-squares using these scaled spectra. The obtained kinetic parameters and concentrations extrapolated to equilibrium were in reasonable agreement with literature values.

### An intelligent algorithm for peptide sequence identification

*Lijuan Hu, Elaine Saulinskas, Peter Johnson and Peter De B. Harrington, Ohio University, Center for Intelligent Chemical Instrumentation, Department of Chemistry, Clippinger Laboratories, Athens, OH 45701-2979*

Peptide sequencing instruments are important tools for bioanalytical chemistry, Knowledge of amino acid sequence permits conclusions to be made about protein structure, relationships among different proteins, and also provides data useful for isolation of the structural gene for a protein. The more common method of N-terminal amino acid sequence determination uses the Edman degradation, in which proteins are successively heated to remove the N-terminal residue which can then be identified by high performance liquid chromatography (HPLC).

The Applied Biosystems 477A Protein/Peptide Sequencer is based on Edman chemistry, and the N-terminal amino acids are identified as their phenylthiohydantoin (PTH) derivatives by HPLC. The PTH-amino acids are identified by their relative retention indices. Incomplete cleavage of the N-terminal amino acids is problematic because the error will propagate through succeeding peptide cleavages and result in background peaks that must be screened from the identification. Indentification of the amino acids may be accomplished by computer algorithms. However, these algorithms are prone to error and frequently require manual interpretation of the chromatographic runs.

An intelligent sequence analysis algorithm has been developed. The algorithm is a fuzzy expert system that uses heuristic rules developed from peptide sequencing experts. The expert system is applied directly to the chromatographic data from the sequencer. This system was comparatively evaluated to an expert and the peptide sequencer onboard software.

### A filter for spectrochemical data with an auto-associative backpropagation neural network

*Busolo Wa Wabuyele and Peter De B. Harrington, Ohio University, Center for Intelligent Chemical Instrumentation, Department of Chemistry, Clippinger Laboratories, Athens, OH 45701-2979*

Artificial neural networks (ANNs) are powerful pattern recognizers that simulate the microstructure of biological nervous systems. A backpropagation neural network (BNN) can be a powerful method for filtering out noise and extracting underlying signals. An autoassociative BNN operates by training the network to perform identity mapping, for which the network inputs are reproduced at the output layer. A spectral filter may be obtained by training the autoassociative network with different spectra of the same chemical composition at the inputs and outputs. By minimizing the number of hidden units the information processed by these units can be maximized in an analogous manner to principal component analysis (PCA). Like PCA, the outputs of the hidden layers can be used to reduce the dimensionality of the data and furnish useful diagnostics for evaluating filter performance.

Laser ionization mass spectrometry (LIMS) is useful for probing failure sites of adhesive bonds. Calibration models may be used on spectra aquired from these sites to ascertain mechanical and chemical properties of the adhesive. Replicate LIMS spectra of epoxy polymers are highly variable. The filter is applied to the LIMS spectra to improve reproducibility. Partial least squares (PLS) regression is used to predict mechanical properties of tensile strength and hardener to resin ratios from the spectra. PLS calibration models were compared between filtered and unfiltered LIMS spectra.

### A new approach to automating the derivatization and trace enrichment of environmental pollutants for HPLC analyses

*Ran Wu, Fran Lai and Landy White, Thermo Separation Products, 45757 Northport Loop West, Fremont, CA 94537*

The use of HPLC for environmental analyses is growing rapidly. There are approximately 10 approved LC methods with 20 or more in various stages of review. The LC procedures are typically very labour intensive, often involving extractions, derivatizations and trace enrichment. A low-cost PC-controlled sample processing workstation is now available that allows the user to easily program sample extractions, derivatizations and even simple solid phase extraction routines for sample cleanup and trace enrichment.

Two of the more common HPLC analyses includes PAHs and aldehydes, in particular, formaldehyde. The analysis for formaldehyde requires DNPH derivatization and both analyses require additional sample cleanup and trace enrichment. Automating these procedures would normally require expensive and complex robotic systems. With the PC control now available on the AS3000 autosampler by Thermo Separation Products, these procedures can be automated at a cost and simplicity consistent with standard HPLC instrumentation.

The setup and use of the new AS3000 autosampler for the automatic derivatization and trace enrichment of environmental analyses were discussed. A comparison of the new automated procedure with the manual methods for PAHs and formaldehyde was reviewed.

### Rapid on-line microwave sample preparation of fatty acid methyl esters for analysis by gas chromatography

*Kathleen E. Williams and Stephen J. Haswell, School of Chemistry, University of Hull, Hull HU6 7RX, UK*

Procedures for the derivatization of fatty acids to their methyl esters for chromatographic analysis are well known to many analysts. Conventionally, these reactions can be time consuming, taking up to several hours depending on sample type and reagents used.

The use of microwave energy to enhance chemical reactions is well documented. The authors' previous experience with microwave FI systems have shown considerable potential for the development of an FI methodology which involves the on-line derivatization of a mixture of saturated and unsaturated fatty acids to their methyl esters for GC analysis. Samples are introduced into an organic carrier stream where they are rapidly derivatised by acid methanolysis under the influence of microwave irradiation and pressure in thin bore tubing. Following derivatization, the methyl esters are precipitated on-line and upon extraction with hexane are analysed as their methyl esters by gas chromatography. The paper discussed the development the preliminary results including the steps through from batch microwave digestion, to analysis of samples by the proposed FI

methodology. The process of derivatization of lipid fractions, such as phospholipids and triglycerides, was also discussed. Results illustrated the compatibility of such reactions for the system described and will discuss the feasibility of interfacing the FI microwave system to a GC for direct analysis.

### Vaporization of liquefied petroleum gases prior to analysis by gas chromatography using a heated pressure regulator sample vaporizer system

*Paul H. Johnson, MTI Analytical Instruments, 41762 Christy Street, Fremont, CA 94538*

A system to vaporize liquefied petroleum gases has been built which converts the liquid mixture into a gas mixture with the same composition as the original liquid. An analytical system suitable for on-line analyses of liquefied petroleum hydrocarbons, flowing in a pipeline or in a process stream, is created when this LPG-sample vaporizer is coupled to a gas chromatograph capable of fast-analyses. The system is also suitable for use in a laboratory environment.

The accuracy of the results produced by this system were verified by comparison to the results obtained by sample loop injection of the liquid under pressure into a conventional gas chromatograph.

This presentation included descriptions of the system components, system performance, and operational considerations such as calibration and maintenance.

### Stepper-motor controlled gas valve inlet system for process GC

*Richard D. Sacks and Mark Nowak, Department of Chemistry, University of Michigan, Ann arbor, MI 48109-1055*

Gas chromatography (GC) is widely used in chemical process monitoring. Its attributes include very high selectivity, wide dynamic concentration range, high sensitivity, and long analysis time. The use of capillary-column GC can result in much shorter analysis times if relatively short columns are used at high carrier gas velocities. However, conventional process-GC inlet systems produce injection band widths which are incompatible with high-speed GC. A high-speed gas valve inlet system controlled by a stepper motor and equipped with a pre-injector for liquid process streams will be described. A continuous stream of sample vapour exists through a small orifice in the side of an electroformed nickel capillary sample delivery tube. This tube passes through a gas tight seal into a pressurized injector block. The injector block contains the capillary separation column and several capillary restrictors used to purge the injection volume. The end of the separation column is located very close to the sample delivery tube with the column axis normal to the sample delivery tube. The stepper motor is used to translate the sample delivery tube so that its orifice is co-axial with the separation column. This results in sample vapour entering the column. After an adjustable time delay, the sample delivery tube is returned to its original position, thus terminating sample injection. By the use of a stepper motor, injection band width and sample size can be controlled by software. Design features and performance data were discussed. High-speed chromatograms of some refinery mixtures were presented.

### On-line monitoring of aromatic hydrocarbons using a fibreoptic UV absorption spectrometer

*T. E. Barber, W. G. Fisher, E. A. Wachter, Health Sciences Research Division, Oak Ridge National Laboratory, Oak Ridge, TN 37831-6113 and R. L. Newmark, Earth Sciences Department, Lawrence Livermore National Laboratory, Livermore, CA 94551*

Numerous fuel spill sites and leaking underground petroleum storage tanks have been identified as potential environmental hazards due to the hazardous nature of aromatic hydrocarbons present in refined petroleum products. Such sites can require extensive cleanup that is both expensive and time consuming. To facilitate cleanup, accurate analytical data on contaminant composition and concentration are required during remediation. In this paper the authors reported on new on-line monitoring techniques that can simultaneously provide both quantitative and qualitative analyses. Specifically, on-line UV absorption has recently been demonstrated at the Lawrence Livermore dynamic stripping site for characterization of aromatic hydrocarbons extracted from a large subsurface pool of petroleum. In the dynamic stripping process, the ground is heated to volatilize and mobilize contaminants. A negative pressure applied at the centre of the contaminant pool efficiently extracts the volatilized materials. Large concentration variations occur during extraction that cannot be feasibly monitored using conventional sampling methods. Since aromatic hydrocarbons have unique absorption spectra in the wavelength region from 210 to 300 nm (while most aliphatic hydrocarbons do not absorb strongly there), UV absorption can be used to completely characterize aromatic hydrocarbon composition of extractant quickly and accurately. A fibreoptic, on-line UV-absorption system was installed at the Livermore site and used to monitor vapour-phase extractant continuously over a period of six-weeks. This presentation detailed the design and operation of the on-line system, discussed the data reduction routines used to interpret the absorption spectra, and compared this technique to other on-line analytical methods.

### Monitoring process reactions by near-infra-red spectroscopy

*Paul H. Dallin and Frank A. Dethomas, NIRSystems, Inc., European Liaison Office, Studio 1, Intec 2, Wade Road, Basingstoke RG24 0NE, UK*

Near-infra-red spectroscopy (NIRS) has developed from a laboratory curiosity to a fully integrated process technique. NIRS is uniquely suited to process analysis as sample preparation is rarely necessary, the technique is totally non-destructive and multiple analyses can be performed in seconds or less.

Robust analysers can be placed in environment ranging from the most benign to hostile at distances ranging from a few inches to a thousand yards from the measurement point by using fibre optics. Intrusion into the process is minimal usually via a small fibre optic probe normally not exceeding 1″ in diameter. These probes are extremely tough, being manufactured from materials such as stainless steel with optical windows of sapphire.

With process measurements both reactants and products can be observed, with results being obtained that allow the identification of developing problems on a time scale that allows corrective action to be taken before any out of specification material is produced, whilst reducing operator exposure to the toxic environment to practically nil. Accurate end-point determination allows an increase in the efficiency with which plant is used, with consequent financial benefits.

In this presentation, the authors reported the monitoring of two chemical reactions of industrial importance and discussed the advantages of real time reaction monitoring that arise from the use of near infra-red spectroscopy. The first reaction studied was the catalytic protonation of a quinone species. In the second reaction a polysaccharide was allylated and the end point was monitored.

### Chemical process-control applications of near-infra-red spectroscopy

*U. Eschenauer, M. Glania, M. Jentzsch, N. Völkl and H. W. Siesler, Department of Physical Chemistry, University of Essen, D 45117 Essen, Germany*

Despite its lack in structural interpretative value near-infra-red light-fibre spectroscopy has become an established technique for chemical process-control. This acceptance is based on the superior information content compared to other less specific on-line methods, the development and improvement of sensors and rapid monochromator/detection systems without mechanically moved parts and the increasingly facilitated implementation in plant environments.

Near-infra-red light-fibre spectroscopy is currently tested in the authors' laboratory for different process-control applications and some selected examples shall demonstrate the potential of this technique. The cases range from monitoring the methylmethacrylate polymerization reaction in tetrahydrofuran solution via the quantitative determination of the phthaloyl content in the production process of a polyester elastomer to the on-line control of physical parameters of poly(ethyleneterephthalate) film in a rheo-optical experiment.

### Portable tools and distributed heterogenous databases in molecular biology

*James M. Ostell, National Center for Biotechnology Information, Bldg 38A, NIH, 8600 Rockville Pike, Bethesda, MD 20894*

Software for biomedical researchers must be able to be quickly developed by scientists as ideas for applications arise. Yet, to be widely used, they must also run on the wide range of computers currently in use, have user-friendly interfaces, and access complex or remotely stored data.

The authors have developed a toolkit for biomedical software development through which a single 'C' language program can be written which will run on Macintosh, IBM PC style, Microsoft Windows, Windows NT, Sun, Silicon Graphics, VAX VMS, DEC ULTRIX, and others, using both a command line or windowing interface as appropriate. Using the toolkit the authors have written production quality software applications which compile and run without change on all supported windowing platforms and which are in wide use by biomedical researchers.

A system of software layers represented by 'C' language libraries provides a flexible robust environment. The CoreLib layer is a thin interface between a single logical view of program flow and operations (on the programmer side) and the details of implementing that view on the various supported platforms. The AsnLib layer, built on the CoreLib layer, provides portable utilities for structured, standardized data exchange using Abstract Syntax Notation 1 (ASN.1, ISO 8824, 8825). The Object layer, built on the AsnLib layer, reads and writes ASN.1 formatted data streams in and out of 'C' structures available to the programmer. The Vibrant layer, built on the CoreLib layer, provides a single programming interface to three different windowing systems. Vibrant supports both a very simple view of user interaction typical of scientific programs and the very complex view of modern highly interactive visual interfaces. An access library supports programmatic data access from CD-ROM, or as Internet client/servers.

The authors created a single formal specification for information relevant to a wide area of biomedical computing, including DNA and protein sequences, genetic maps, physical maps, the biomedical literature, and others, using ASN.1. They have developed a suite of software tools for creating, exploring, and manipulating data in this form. A number of important databases have been converted to this unified data model and provide integrated access to this heterogenous system on CD-ROM and over Internet.

### Current status of the laboratory automation standards foundation: programs and advancements

*Joseph G. Liscouski, LASF, PO Box 38, Groton, MA. 01450*

The LASF is a non-profit organization designed to advance the practice of laboratory automation. Over the past two years it has grown from an idea to a functioning organization actively participating in identifying and solving problems in its field.

Among its efforts are:

(1) The development of new computing models—the LASFs laboratory model emphasizes the process characteristics of laboratory work.

(2) Identification of needed technologies—as a result of the computing models development, LASF has been able to define new technologies and products that will improve lab automation.
(3) Educational courses and symposia—in addition to successful courses on strategic approaches to laboratory computing, data acquisition, databases, etc. the LASF has begun holding symposia on the validation of laboratory systems. It has organized a working group to document industry practices in the validation of instruments, data systems, and LIMS.
(4) A project to develop data format standards for atomic spectroscopy techniques.

This presentation reviewed the work done, and the direction that the organization is taking in the future.

### ASTM E-31 standards for clinical and analytical laboratory automation

*Robert Megargle, Department of Chemistry, Cleveland State University, Cleveland, OH 44115*

ASTM Committee E-31 on Computerized Systems has been working for many years to develop standards for automation relevant to chemical laboratories. Voluntary consensus standards can be useful to promote common terminology, accepted logical structure for information and knowledge, consistent practices and test methods, and better connectivity between separately designed equipment. Standards are the key to reducing the problems and chaos that many designers face when building automated systems. This presentation dealt with the current status of ASTM E-31 standards for clinical and analytical laboratories. Included were specifications for connecting clinical instruments to Laboratory Information Systems, standard message content for transferring clinical information between independent computer systems, procurement guidelines for automated laboratory instruments and information systems, system implementation strategies, standard bar code labels on clinical specimen containers, system documentation specifications, and procedures for maintaining reliability records of clinical computer systems.

### Raman spectroscopy—academic laboratory to the process

*Arlene A. Garrison, Madhavi Z. Martin and Michael J. Roberts, Measurement and Control Engineering Center, University of Tennessee, 102 Estabrook Hall, Knoxville, TN 37996-2350*

Research at the University of Tennessee, Knoxville has led to the development of an on-line Raman spectrometer for chemical composition determination. The on-line spectrometer provides real-time composition information from an internal stage of a distillation column at the Eastman Chemical Company in Kingsport, Tennessee.

The Raman analyser uses an air-cooled ND:YAG laser and a Fourier transform infra-red spectrometer modified to operate in the near infra-red region. Silica optical fibres allow remote measurements by transmitting the laser radiation to the process. Raman scattered light is collected by six fibres and returned to the analyser in the control room for spectroscopic analysis and further mathematical manipulation.

The sensor development project has received funding from the Department of Energy and the Measurement and Control Engineering Center, an NSF Industry/University Cooperative Research Center. Several steps were essential to this five year development. Feasibility studies established the utility of Fourier Transform Raman for the specific problem of distillation column analysis. Fibre-optic interfacing and instrument modifications were necessary to provide an acceptable plant floor instrument. Mathematical models of the distillation column were used to determine optimum sensor placement, critical to application of any on-line device. Several data analysis methods were investigated to determine the method with the greatest accuracy and robustness. The results and conclusions from the recently completed field test were discussed. Research progress on fibre-optic based probes and studies related to other potential applications for FT-Raman in industrial environments were reviewed.

### Evaluation and application of 'hand-held' gas chromatography for refinery air monitoring

*P. A. David, Amoco Corporation, PO Box 3011, Naperville, IL 60566-7011 and S. A. Ness, CIH, Amoco Oil Company, Whiting Refinery, 2815 Indianapolis Blvd., Whiting, IN 46394-0710*

Successful transfer of new analytical technology from the laboratory to the plant requires a team approach. The partnership forged for the evaluation and implementation of hand-held gas chromatography for refinery air analysis consisted of research and development, refinery, and vendor representatives. The functions of this partnership for the utilization of hand-held gas chromatography for airborne benzene determination will be illustrated for critical refinery health and safety applications such as confined-space entry and 'real-time' personnel exposure monitoring.

Technology transfer from the laboratory to the plant generally requires, (1) an understanding of the analysis problem, (2) evalution of a reliable commercial instrument, (3) laboratory and field evaluation of this product, and (4) routine utilization of this technology by plant personnel. This process is evolutionary in many ways because the development and implementation of the technology is fostered through the communication between users and the vendor throughout the problem definition and evaluation stages.

This presentation discussed the analytical and safety requirements of the refinery airborne benzene determination by hand-held gas chromatography. The results of laboratory and field evaluations of the instrument were presented emphasizing their impact on instrument evolution. Additionally, the protocol for routine implementation of hand-held gas chromatography by refinery personnel were outlined.

### Supercritical fluid chromatography from the laboratory to the process

*Jerry M. Clemons, ABB Process Analytics, 843 North Jefferson Street, Lewisburg, WV 24901*

The Supercritical Fluid Chromatograph (SFC) has the ability to do a number of analyses not possible with Gas or Liquid Chromatographs and, in many cases, SFC can provide the analysis much quicker than a GC or LC. Supercritical Chromatography can be used for samples that are higher in molecular weight, have higher boiling points, and are thermally labile (unstable). Because of this innate ability the SFC is ideal for many of the measurements encountered in a refinery.

These measurements include boiling point distribution or simulated distaillation of some of the heavier fractions from the atmospheric tower section of a crude unit. From this section the middle distillätes, heavy atmospheric gas oil, and vacuum gas oils are fractions which are ideally handled with the SFC. Another application for which this analyser is ideally suited is on the feed to the crude unit after the desalter. This location will provide a boiling point distribution on the crude to assist in the optimization of fraction cuts from the tower.

The sample is taken from the feed line and passes through the sample handling system to remove the fine particulates and provide the flow necessary for the analyser. From the sampling system the sample passes into a diluter value on the SFC where a few microlitres are diluted with supercritical carbon dioxide, this dilution is appromately 500:1 and eliminates the need to use a splitter on the inlet of the SFC. One concern with this approach involves the asphaltenes which are not soluble in supercritical carbon dioxide. The residue which remains undissolved in the valve is flushed out on a periodic basis using a suitable solvent. This can be accomplished on a daily or weekly basis depending on the concentration remaining. These are the sample components of the sample which end up in the vacuum bottoms section of the tower and, thus, have minimal effect on the boiling point distribution and, in turn, the optimization process.

The diluted sample passes out of the diluter to the sample injection valve where a 0·2–1 μl sample volume is injected onto a capillary column. This fused silica column, 10–20 metres in length, separates the components of the sample by boiling point prior to their passing into a step-down restrictor and the detector for measurement. The step-down restrictor allows the carbon dioxide carrier in the column to be maintained in a supercritical state across column and prior to passing into the detector. As the carrier and sample elute from this restrictor they drop to atmospheric pressure. Finally, the sample plus carrier flow into the flame ionization detector for measurement. The detector is maintained at a temperature above 300°C to prevent condensation and ensure optimal operation.

This is an ideal application for a Supercritical Fluid Chromatograph to provide an important parameter, boiling point distribution, for optimal control of the tower. This analysis is very important when the feed to the tower varies frequently requiring routine adjustments of the side cuts to obtain the most economic operation. Another ideal application is the feed to the Fluid Catalytic Cracking Unit (FCCU).

### New NIR technology for process analysis: A collaboration between academia, industry and instrumentation manufacturer

*John Coates, David Tracy and Jerome Workman, Perkin–Elmer Corporation, Real-Time Systems Division, 761 Main Avenue, M/S 201, Norwalk, CT 06859-0201*

Several university-based research centres, such as CPAC (Center for Process Analytical Chemistry) have been established during the past decade. Such centres have in part been funded by industrial sponsors, which for the most part has included most of the major chemical and petroleum manufacturing companies. The main concern of sponsoring companies has always been the realization/ applicability of the 'products' of funded research projects, and the ability to gain practical, demonstrable results from the sponsorship—an intangible when attempting financial justification to upper management.

In recent years, the Federal government has realized the importance of intellectual resources, and is actively working on methods for transferring technology from the research centres, including university research centres, into industry. Technology can take on many forms, and in a case as CPAC, this can include 'know-how', analytical methods, computer hardware and software, and instrumentation concepts. Many of these may be implemented by sponsors by absorption of the ideas/techology into an existing R & D structure. The latter issue, however, is much harder to implement. The ideal is to be able to transfer the technology into commercialized instrumentation, thereby making it readily accessible, as an off-the-shelf item. In most cases this requires direct involvement of an instrument manufacturer.

For an instrument manufacturer, some of the issues are: the costs of development, the return on investment, the size of market, and the general applicability of a product once manufactured (i.e. does every system require customization?). Also, another aspect is the actual transition from an R & D concept to a final manufactured instrument. It is important that the final product meets the needs of the end-user. The optimum way of handling this situation is to involve the end-user in the product development. This paper addressed such a situation, where a concept was conceived in a university R & D group, and was subsequently transferred into industry with the production of a commercial process near-infra-red analyser. In this particular example, the original proof of concept was developed by CPAC, and was taken through feasibility and field-test phases to the final form by two CPAC sponsors—Amoco and Perkin–Elmer.

### Desorption efficiencies of air samples collected on thermal desorption tubes

*Scott A. Hazard and Jamie L. Brown, Supelco, Inc., Supelco Park, Bellefonte, PA 16823-0048*

When analysing air samples collected on thermal desorption tubes, the amount of analyte that is thermally removed from the tube to the gas chromatograph during the desorption process, is very important. The higher the desorption efficiency, the more accurate measurements of analytes will be.

Several thermal desorption tubes containing various combinations of adsorbents were spiked with volatile organic compounds (VOCs) commonly listed in USEPA air sampling methods. Inert gas was passed through the tubes to simulate typical collection volume used in air sampling.

The effect of different sample volumes on desorption efficiencies for specific analytes from different chemical classes was measured and was discussed in this paper.

### The automated injection of large sample volumes in capillary gas chromatography using PTV injection techniques

*Anne S. Williams, Valerie Lopez Naughton, Tekmar Company, PO Box 429576, Cincinnati, OH 45232-9576 and Peter Ridgeon, Ai Cambridge, London Road, Pampisford, Cambridge CB2 4EF, UK*

Most injection techniques used in capillary gas chromatography (GC) are limited to sample volumes of a few microlitres. This restricts the limit of detection the GC system is capable of achieving.

Programmed Temperature Vaporizing (PTV) Injectors offer the ability to introduce sample volumes which are greater than previously possible. Recent developments have enabled sample injection to be fully automated and used with most modern gas chromatographs.

The paper discussed the technique of large volume sample introduction and the various parameters which effect its performance. It also gave a number of examples of its application to real life problems where significant improvements in detection limits can be achieved.

### A computer based model for optimizing speed, sensitivity and minimum detectability of capillary column separations

*Richard Villalobos. OnLine Analytics, Box 1742, Duxbury, MA 02331-1742*

OnLine GC Lab, a PC-based software package for optimizing gas chromatography columns, quickly finds the best operating conditions to give the fastest separation with the best sensitivity. The model can be used to design column systems with a single phase or multidimensional systems that use series-coupled columns with dissimilar phases. Based on the modified Golay–Giddings equation, the model permits a rapid manipulation and evaluation of column variables (such as diameter, length, film thickness) and operating conditions (temperature and carrier type and flow rate) to evaluate a column 'in hand' or to determine the 'best' column to purchase.

Especially designed for fast, on-line chromatographic analyses, the model is particularly useful for optimizing high-speed separations for process control while satisfying user specified minimum detectability and resolution of key sample components. An advanced algorithm takes into account the time constant of the detection system to tailor the application to a particular chromatograph of known characteristics.

An integral data base of thermodynamic-based retention indexes permits selection of the best phase for a given separation, and determines the operating conditions that best satisfy the analytical requirements. The data base can be augmented by the user with in-house experimental data for continued growth of its usefulness and applicability.

Calculated optimum conditions are presented in tabular form (retention times and peak widths) or as chromatograms in the graphics mode. The software package also includes window diagrams for determining the optimum ratio of phases in multidimensional systems, and other utilities for quickly converting laboratory retention data to thermodynamic-based retention indexes for adding to the data base.

### The NIST consortium on automated analytical laboratory systems (NIST CAALS) standards for automated systems used in analytical chemistry

*Gary W. Kramer, National Institute of Standards and Technology, Chemical Science and Technology Laboratory, Chemistry Building 222, M/S A-213, Gaithersburg, MD 20899*

The Consortium on Automated Analytical Laboratory Systems (CAALS) is a partnership between companies from the private sector and government agencies and is hosted by the National Institute of Standards and Technology (NIST). CAALS' overall goal is to foster the development of automation in analytical chemistry.

CAALS has undertaken, with guidance from its members and others in the analytical instrumentation community, the task of identifying, defining, and promoting general guidelines and standards in critical areas of sample, data, and control information interchange for analytical instruments. The Consortium has developed a protocol and syntax specification for communications between instruments and controllers (CAALS-I). This specification built from existing standards and common practices is independent of computing platforms and provides for guaranteed message delivery and connectivity across several common physical links. The specification is now undergoing an external review, and we expect the initial version of the formational specification document will be available early in 1994. In addition to the formal specification, CAALS is developing specific implementations of CAALS-I on a variety of computing platforms. Since it is our hope that such applications will be useful to others in implementing the specification for their own use, we will provide these examples as addenda (complete with source code) to the formal document. Finally, we are preparing a controller-end conformance tester for verifying the correct operation of instruments using the CAALS-I specification. This presentation described progress to date.

### NIST CAALS: behaviours for instrument controllability

*Marc L. Salit, National Institute of Standards and Technology, Chemical Science and Technology Laboratory, Chemistry B-222, Gaithersburg, MD 20899 and J. Michael Griesmeyer, Sandia National Laboratories, Intelligent Machine Systems Division, Albuquerque, NM 87185*

This paper described a set of device behaviours which facilitate the construction of reliable, safe and efficient automated systems. The classic approach to automated system development has relied on the acquisition of a group of devices, together which provide the appropriate capabilities to perform the system job. These devices are then 'integrated', or made to work together to effect the desired job. How this 'system integration' is performed has critical impact on the ultimate cost, utility, reliability, safety and efficiency of the automated system.

Lessons learned from the experience of a group of system integrators has allowed the authors to identify several fundamental characteristics of easily integrated devices. These characteristics can be expanded into groups of behaviours, derived from the fundamentals, which can be used to identify and classify devices which are to be used as subsystems in an automated system.

### An overview of step: the STandard for Exchange of Product model data

*Joseph A. Carpenter, Jr., Building 223 (MATL), Room A256, National Institute of Standards and Technology, Gaithersburg, MD 20899*

This presenatation described and updated the status of STEP (STandard for Exchange of Product Model Data), an emerging world standard for the computerized exchange of data on manufactured products.

STEP is an outgrowth of the Initial Graphics Exchange Specification (IGES) which, while highly successful, was limited primarily to exchange of only graphical data needed by CAD/CAE/CAM practitioners to physically describe a product. STEP, on the other hand, attempts to focus on ALL data on a manufactured product, from conceptual design, through manufacture, to in-service performance and even disposal. STEP attempts to do this by providing standard, neutral exchange formats through which, conceivably, any STEP-compatible operating programs or databases can communicate with one another. After almost ten years of development, the International Organization for Standardization (ISO) has released the first elements of STEP and various groups around the world are beginning implementations of it in industrial activities.

### Synchronous luminescence: From field screening of hazardous waste to laser-induced technique for human health protection

*Tuan Vo-Dinh, Advanced Monitoring Development Group, Oak Ridge National Laboratory, Oak Ridge, TN 37830-6101*

This presentation provided an overview of the various applications of the simple luminescence technique based on synchronous excitation ranging from process control to human health monitoring.

The synchronous luminescence (SL) methodology is a simple way to measure the luminescence signal and spectral fingerprints for rapidly screening complex chemical samples. Conventional fluorescence spectro scopy uses either a fixed-wavelength excitation to produce an emission spectrum or a fixed wavelength emission to record an excitation spectrum. With synchronous spectroscopy, the fluorescence signal is recorded while both are simultaneously scanned. A constant wavelength interval is maintained between excitation and the emission monochromators throughout the spectrum. A portable battery-operated SL instrument has already been developed for field monitoring. The device was used to screen hazardous wastes for organic chemicals or to detect gasoline leaks from storage tanks. Another SL system using tunable laser excitation was developed for sensitive detection of chemicals (subatomole detection). A variety of applications of the SL methodology were presented, from real-time environmental monitoring, petroleum process control, DNA sequencing applications to human health protection (DNA adduct detection).

### Infra-red monitoring: A case study of emission control systems and process streams

*Jess S. Eldridge, Gary D. Hipple, William K. Reagen, Jeffrey W. Stock, 3M Environmental Laboratory, 935 Bush Ave., Building 2-3E-09, St. Paul, MN 55133-3331 and Martin L. Spartz, Department of Chemistry, Central Michigan University, Mt. Pleasant, MI 48859*

Infra-red spectrometers have been used to monitor the release of volatile compounds from emission control systems and process streams at various 3M facilities. Infra-red monitoring allows for near real time collection of data for a wide range of processes and systems. Real time monitoring for fluctuations in component emissions can provide insight on how to reduce chemical emissions and provide on-line information necessary to optimize process variables.

Extractive FTIR measurements incorporated either a 10-metre multiple reflection or a 10-centimetre single pass IR gas cell. Less than 32 coadded scans were collected so dynamic systems could be rapidly monitored for changes. All FTIR measurements of process gases were acquired in conjunction with testing by approved methods to determine the levels of data correlation.

Extractive FTIR application presented included a study to measure ppmv (parts per million by volume) process levels of methanol and toluene, a catalytic oxidizer emission study conducted to establish process variable optimization, and a study to measure low ppmv levels of styrene in a drying oven system of a polystyrene film processing line.

### On-line transient infra-red analysis of polyethylene encapsulation nuclear waste streams

*Steven L. Wright, Roger W. Jones and John F. McClelland, Ames Laboratory—USDOE, Iowa State University, Ames, IO 50011*

Transient infra-red spectroscopy (TIRS) is a technique that has been developed to obtain useful transmission or emission spectra of moving optically thick solids and viscous liquids. Currently, this technique is being developed for monitoring the polyethylene encapsulation of low-level radioactive waste. In the encapsulation process, radioactive-salt waste is mixed with polyethylene pellets, heated, and extruded as a molten stream. Upon cooling, the mixture solidifies to a monolithic waste form with excellent properties for long-term storage.

A comparison of the relative merits between the transmission and emission modes of TIRS were presented. Various experimental parameters, such as linear velocity and temperature of the molten stream were also discussed.

### Speciation of organometallic compounds (Sn, Hg) with cryofocusing and detection by ICP/MS

*Christophe Quetel[1], Fabienne M. Martin[1], Riansares Munoz[1], Francis Grousset[2] and Olivier F. X. Donard[1], [1] Laboratoire de Photophysique et Photochimie Moléculaire, Université de Bordeaux I, 33405 Talence, France, [2] Départment de Géologie et Océanographie Université de Bordeaux I, 33405 Talence, France*

The determination of organotin and organomercury compounds in the environment is of great concern due to the high toxicity of these species. Most techniques developed to date have used most frequently a gas chromatographic separation approach with various types of detectors. These species are classically derivatized with $NaBH_4$ or $NaBEt_4$. They are then trapped in liquid $N_2$ on a small packed chromatographic column. After the trapping stage which allows efficient preconcentration of the analytes, the volatile species are sequentially introduced in the detector upon warming of the trap.

In this work, the detector is ICP/MS. Such sample introduction combines several optimizing conditions for ICP/MS sample introduction. The analytes are separated from the matrix minimizing later potential interferences. The analytes are preconcentrated and introduced in the gaseous state allowing 100% sample introduction. These conditions allow to reach high sensitivity. The authors described the general results and improvement of sensitivity obtained with such sample introductions in ICP/MS and discussed its limitations.

### Non-invasive determination of tablet formulation through a plastic bottle using short wavelength near infra-red spectroscopy

*Anna G. Cavinato, David M. Mayes, DSquared Development, Inc., 1108 J Ave., LaGrande, OR 97850 and Paul K. Aldridge, Pfizer Central Research, Eastern Point Rd, Groton, CT 06340*

Determination of tablet formulation can be accomplished by using diffuse reflectance and diffuse transmission techniques in the Near Infra-red spectral region. Recent developments indicate that this technique can be extended to qualitatively determine tablet formulation of samples already packaged and sealed in a typical 100 tablet plastic bottle. The technique utilizes Short Wavelength Near Infra-red spectroscopy (SW-NIR: 700–1100 nm) to analyze light from a standard 10-watt tungsten bulb transmitted through the entire thickness of the bottle and enclosed tablets. A study of several different tablet types and formulations was illustrated using standard chemometric classification techniques. This technique could be used in conjunction with a bar code scanner to verify a specific tablet formulation is labelled correctly during packaging.

### In-process particle size distribution measurements and control

*Thomas L. Harvill and Donald J. Holve, Insitec, Inc. 2110 Omega Road, San Ramon, CA 94583*

In-process measurement of particle size and concentration distributions provides continuous analysis and quality control of a product stream and can be used to monitor particulate emissions. As process production rates continue to improve, the delay between laboratory analysis and process correction of the product stream become more significant and costly in many applications. Elimination of sample handling and operator manipulation is now possible for most pneumatic flows using optical methods which are properly interfaced with the process stream. Insitec has developed a range of laser-optical instruments for application in difficult environments. One of these instrument techniques has shown significant capabilities for powder manufacturing industries.

The EPCS-Eductor (Ensemble Particle Concentration & Size-Eductor) has been used to obtain detailed size distribution measurements in powder production facilities at two second intervals. This fast data acquisition rate and display allows for real-time particle classification control on any user-selected element of the size distribution. Recent results for pharmaceutical powders, paint coatings, and toners were presented.

### Monitoring nitroglycerine in transdermal patches by near-infra-red spectroscopy

*Denise E. Grzybowski and Stephen L. Monfre, NIRSystems, Inc., 12101 Tech Road, Silver Spring, MD 20904*

Transdermal patches are used to transfer doses of nicotine through the skin to a smoker trying to quit, and medications, such as nitroglycerine, for persons with heart problems. These patches are used for the time released dosage mechanism which allows the user to receive the drug at a steady rate. Monitoring the amount of drug present on the patch is essential to assuring the safety of the patient as well as the integrity of the product. Near-Infra-red (NIR) spectroscopy is a non-destructive technique which provides results in less than one minute thus allowing analysis of multiple patches per lot of material.

The detection of nitroglycerine in transdermal patches by NIR spectroscopy will be described in this presentation. Samples which varied in both active concentration and applicator pump speed were incorporated into one calibration model for the purpose of monitoring drug weight per patch. The presentation focused on the effect different pump speeds appear to have on the accuracy and precision of the NIR measurement. Discussions included whether NIR analyses can be incorporated into the process, atline, or in the Quality Control laboratory. The advantages of each analysis approach was presented along with their disadvantages. A conclusion was drawn as to what changes in instrumentation would be required to make real-time analysis possible.

### Material sampling: The key to near-infra-red analysis

*Denise E. Grzybowski, NIRSystems, Inc., 12101 Tech Road Silver Spring, MD 20904*

Near-infra-red (NIR) analysis is now becoming a widely accepted quantitative analysis tool in industries other than agriculture. In the agricultural industry, samples are obtained, prepared (often involving the grinding of solid samples into a powder) and analysed using a variety of near-infra-red technologies (filters, gratings, diode-arrays). In other industries, such as the polymer, textile, and pharmaceutical industries, this simple sample preparation is not feasible. Samples from these industries include polymer pellets, textile bobbins, intact tablets, and transdermal patches.

Due to the large variety of samples analysed by NIR, this presentation focused on the types of sampling methodologies available with NIR instrumentation. These sampling methodologies range from sample cups and cuvettes to fibre-optic reflectance and transmission probes. Guidelines for determining the appropriate sampling methodology for different applications were provided as was discussion on the limitations of each sampling method. The effect that each method has on the overall accuracy and precision of the NIR measurement was also presented.

### Remote monitoring of sub PPB levels of vinyl chloride, dichloroethylene and trichloroethylene via modem operated automated GC

*Dr. Amos Linenberg and Neil J. Lander, Sentex Systems Inc., 553 Broad Avenue, Ridgefield, NJ 07657*

The need for remote monitoring of certain compounds led to the development of a portable gas chromatograph remotely operating through a modem. The compounds being monitored were Vinyl Chloride, Cis 1,2 Dichlorethylene and Trichloroethylene. The requirements of the project were to detect these compounds at the ppb level in air.

The GC was controlled by a lap-top computer. The operation of the instrument was as follows:

(1) An internal pump pulled the air in and onto a preconcentration device.
(2) The VOCs were then thermally desorbed from the preconcentrator.
(3) Carrier gas swept the VOC's on to the column for separation and detection.
(4) All data was stored automatically to the hard drive of the computer.

The operating parameters were as follows:

Sample Time: 250 seconds, Columns: 4 foot 1% SP1000 on Carbopak B, Oven Temperature: 90°C, Carrier Gas Flow: 20 cc/mn, Detector: Argon Ionization Detector, Preconcentrator was packed with 60/80 Carbosieve G.

The instrument was calibrated periodically with a certified mixture supplied by Sctott Specialty Gases. The actual calibration concentration of the analytes in the calibration mixture was 1·15 ppb VC, 1·10 ppb DCE and 1·09 ppb TCE.

The remote instrument was located in the midwest portion of the US. It was periodically contracted by the main computer, located in northern New Jersey, for review and correction of any unexpected variation in instrument responses. The data was uploaded weekly from the remote instrument to the main computer in New Jersey and then reformatted for distribution to the client.

### Analysing data from *in-situ* monitoring with fibre-coupled IR probes

*R. E. Aries, D. J. Cutler, D. P. Lidiard, and R. A. Spragg, Post Office Lane, Beaconsfield, Buckinghamshire HP9 1QA*

FT-IR spectra can be measured rapidly and are sensitive to a wide variety of chemical and physical changes. With the availability of mid-IR fibre-coupled probes it is now practicable to measure the spectra of liquids without transferring them to a special cell. This has opened up the possibility of using FT-IR as a convenient and versatile monitor to follow reactions or equilibria. However, it may be difficult to find absorption bands for different species which are free from overlap, and indeed it may not be possible to isolate some species involved in order to obtain their spectra. This presents a considerable problem since quantitative analysis is needed. In this paper the authors showed how multivariate analysis can provide a solution.

Usually it is not possible to determine the spectra and concentrations of the components in a series of mixtures when neither are known. Principal component analysis can be used to reduce the spectra to a number of factors, corresponding to the number of species present. The concentration of each species is given by an unknown combination of the factor scores. It becomes possible to find these combinations if there is a model which the concentrations must fit. For example in an equilibrium between two species it is possible to find that value of the equilibrium constant which provides the best fit to concentrations derived from the measured factor scores. The optimization procedure determines both the equilibrium constant and the spectra of the individual species. Kinetic data can be treated in the same way.

The method was illustrated with data obtained with both mid-IR ATR and NIR transmission probes. It uses information from the whole spectrum and so is both more sensitive and more widely applicable than methods which are restricted to individual bands.

### *In-situ* chemical characterization of waste sludges using FTIR-based fibre optic sensors

*Teofila V. Rebagay, David A. Dodd, Larry L. Lockrem, David W. Jeppson and Gordon R. Blewett, Westinghouse Hanford Company, Richland, WA 99352*

The characterization of unknown mixed wastes is a mandatory step in today's climate of strict environmental regulations. Cleaning up the nuclear and chemical wastes that have accumulated for 50 years at the Hanford Site of the US Department of Energy near Richland, Washington is the largest single cleanup task in the United States today. The wastes are stored temporarily in carbon steel single- and double-shell tanks at the site. Their final disposal depends on accurate analyses of their chemical and radiochemical compositions.

This study is focused on near-infra-red (NIR) and mid-infra-red (MIR) fibre optic sensors interfaced to a Fourier transform infra-red (FTIR) spectrometry system for *in-situ* measurement of the chemical composition of Hanford Site waste sludges. Silica and silver halide optical fibres were used for the NIR and MIR sensors, respectively. The FTIR-based NIR sensor appeared to be very attractive for the fast and direct determination of the water content of the wastes while the MIR sensor may be employed to measure the concentrations of cyanoferrates and oxyanions of interest. The analytical utility of these sensors to analyse highly radioactive waste sludges was discussed.

### *In-situ* monitoring of organics in aqueous solutions by evanescent wave spectroscopy

*Dianna S. Blair and Lloyd W. Burgess, Center for Process Analytical Chemistry, University of Washington, Seattle, WA 98195*

Evanescent wave spectroscopy (EWS) is a promising technique that combines the convenience of fibre optics with the quantitative and qualitative aspects of infra-red spectroscopy. Based on the principle of attenuated total reflection (ATR), EWS is inherently not a very sensitive technique due to the small amount of energy present in the evanescent field. To increase the sensitivity of EWS to various organic analytes in aqueous solutions plastic clad silica fibres (PCS) were used as EWS elements. It was observed that the polymer cladding provides a matrix into which the organic species can partition, concentrating the organics in the evanescent field and, therefore, increasing the derive sensitivity.

Partition coefficients for various aqueous organic analytes into two commercially available polymer clad fibres was calculated based on the evanescent spectra of analyte in the cladding, at equilibrium, and depth of penetration of the evanescent field. A dimethylsiloxane polymer cladding yielded partition coefficients for aqueous toluene, 1,1,1 trichloroethane, and trichloroethylene of 11, 27, and 45, respectively. Reduced partitioning was observed for a fluorinated silicone clad fibre resulting in partition coefficients of 6, 16, and 28, for the above analytes, respectively.

Detection limits for these analytes were determined based on a 0·9 m length of the two fibres studied and are listed below.

| | Detection limits | |
|---|---|---|
| | Dimethylsiloxane clad | Fluorinated silicone clad |
| Toluene | 17 ppm | 32 ppm |
| Trichloroethane | 21 ppm | 35 ppm |
| Trichloroethylene | 5 ppm | 12 ppm |

These results suggest that for some applications this format may be useful for detecting and quantifying aqueous organic compounds.

### Noninvasive near-IR/ARS/MHD analysis of pharmaceuticals

*Robert G. Buice, Jr and Robert A. Lodder, University of Kentucky College of Pharmacy, Lexxington, KY 40536-0082*

There have been instances of illness and injury due to pharmaceuticals that were either mislabelled or failed to dissolve as expected. Several methods were introduced that can be used as online process control assays to differentiate between different pharmaceuticals and also distinguish between pharmaceuticals that pass and fail dissolution tests. Near-IR reflectance spectroscopy has been used to analyse several pharmaceuticals (tablets and capsules) and the dissolution times have been correlated to the near-IR spectra with minimal error.

Near-IR can also be used to differentiate liquid pharmaceuticals. The major drawback to near-IR analysis is the fact that the results correlate to the composition of the surface of solid pharmaceuticals. In coated capsules and tablets better differentiation has been achieved using Acoustic Resonance Spectroscopy (ARS) which confers information about the bulk properties of the pharmaceutical (density, adsorbed water etc.).

The same predictions of dissolution rate with ARS and near-IR have been established. Magneto-Hydrodynamic Spectroscopy is accomplished by coupling a magnetic field with the acoustic wave from ARS to yield information about the ionic composition of the pharmaceutical. The authors have also used an InSb focal plane array camera to detect water uptake in capsules. PCA can be used to show degradation products and to allow the construction of kinetic models for the formation of degradation products.

